# Mitochondrial-bacterial hybrids of BamA/Tob55 suggest variable requirements for the membrane integration of β-barrel proteins

**DOI:** 10.1038/srep39053

**Published:** 2016-12-16

**Authors:** Anna-Katharina Pfitzner, Nadja Steblau, Thomas Ulrich, Philipp Oberhettinger, Ingo B. Autenrieth, Monika Schütz, Doron Rapaport

**Affiliations:** 1 Interfaculty Institute of Biochemistry, University of Tübingen, 72076 Tübingen, Germany; 2 Interfaculty Institute of Microbiology and Infection Medicine, University of Tübingen, 72076 Tübingen, Germany

## Abstract

β-Barrel proteins are found in the outer membrane (OM) of Gram-negative bacteria, chloroplasts and mitochondria. The assembly of these proteins into the corresponding OM is facilitated by a dedicated protein complex that contains a central conserved β-barrel protein termed BamA in bacteria and Tob55/Sam50 in mitochondria. BamA and Tob55 consist of a membrane-integral C-terminal domain that forms a β-barrel pore and a soluble N-terminal portion comprised of one (in Tob55) or five (in BamA) polypeptide transport-associated (POTRA) domains. Currently the functional significance of this difference and whether the homology between BamA and Tob55 can allow them to replace each other are unclear. To address these issues we constructed hybrid Tob55/BamA proteins with differently configured N-terminal POTRA domains. We observed that constructs harboring a heterologous C-terminal domain could not functionally replace the bacterial BamA or the mitochondrial Tob55 demonstrating species-specific requirements. Interestingly, the various hybrid proteins in combination with the bacterial chaperones Skp or SurA supported to a variable extent the assembly of bacterial β-barrel proteins into the mitochondrial OM. Collectively, our findings suggest that the membrane assembly of various β-barrel proteins depends to a different extent on POTRA domains and periplasmic chaperones.

Membrane-embedded β-barrel proteins are exclusively found in the outer membranes (OM) of Gram-negative bacteria and eukaryotic organelles directly derived from prokaryotic ancestors namely, mitochondria and chloroplasts. Although most of the proteins in the bacterial OM are members of this protein class, only five mitochondrial β-barrel proteins were identified in yeast so far^1^.

In bacteria, β-barrel proteins are synthesized with N-terminal signal sequences and upon their appearance at the ribosomal exit channel they can be stabilized by trigger factor. Subsequently, the chaperone SecB is proposed to bind the nascent polypeptide chain and to direct it to the Sec translocon that mediates translocation of the substrate protein across the inner membrane^2^. Reaching the periplasm, the signal peptide is cleaved off and the precursor protein is escorted towards the OM by chaperones. The precise roles of the chaperones SurA, Skp and DegP are still debated and seem to differ depending on substrate and organism^3^,^4^. These chaperones relay the substrate protein to the β-barrel assembly machinery (BAM) that resides in the OM and facilitates the actual membrane assembly of the β-barrel protein. In *Escherichia coli*, this complex is composed of the central β-barrel protein BamA and its four associated lipoproteins (BamB, BamC, BamD and BamE)^5^,^6^,^7^. Despite remarkable progress in characterizing factors involved in the biogenesis pathway of β-barrel proteins, the exact mechanism by which these proteins are assembled into the membrane still remains unresolved.

In eukaryotic cells the precursors of mitochondrial β-barrel proteins are synthesized on cytosolic ribosomes and subsequently are recognized via a β-hairpin motif at the organelle’s surface by import receptors of the translocase of the outer membrane (TOM complex)^8^. Then they are transferred across the OM through Tom40, the general entry gate of the complex, a β-barrel protein itself^1^,^9^,^10^. Within the intermembrane space (IMS) the precursor proteins are protected from aggregation by the hetero-hexameric Tim chaperone complexes Tim8/13 and Tim9/10. Assembly of the precursor proteins into the OM occurs via a dedicated protein complex termed topogenesis of outer membrane β-barrel proteins (TOB) complex or sorting and assembly machinery (SAM)^11^,^12^,^13^,^14^. This complex is composed of the central conserved β-barrel protein Tob55/Sam50 and the peripheral subunits Tob38/Sam35 and Mas37/Sam37 that are located on the cytosolic side of the membrane.

During the endosymbiosis process, the developing mitochondria had to adapt in order to ensure import of proteins from the cytosol. Nevertheless, functional expression of bacterial β-barrel proteins in eukaryotic cells suggests that during this adaptation process the ability of mitochondria to facilitate the assembly of prokaryotic β-barrel proteins was maintained^15^,^16^,^17^,^18^. A closer look at the biogenesis pathways of β-barrel proteins reveals that many characteristics are shared among Gram-negative bacteria and mitochondria. First, in both cases the precursor proteins are initially translocated across a membrane and prevented from aggregation by specialized chaperones. Second, insertion into the OM occurs from the inner side of the membrane. Third, the most striking similarity is the sequential and functional homology in the central components of the assembly machineries, Tob55 and BamA, both being members of the Omp85 superfamily^19^. Apart from the aforementioned similarities, the assembly process differs in terms of accessory proteins. Whereas in mitochondria, the two accessory subunits are located at the cytosolic side of the OM, all accessory lipoprotein subunits of the BAM complex are exposed to the periplasm. Moreover, the mitochondrial accessory subunits lack sequence similarity to the auxiliary subunits of the bacterial BAM complex^20^.

All proteins belonging to the extended Omp85 superfamily are predicted to be composed of one or more polypeptide-transport-associated (POTRA) domains in their N-terminal portion followed by a 16-stranded C-terminal β-barrel domain. Indeed, recent structures of BamA and the whole BAM complex confirmed this arrangement^7^,^21^,^22^. Although the primary-sequence similarity of the POTRA domains is low, structures revealed that the folds in each domain are similar, comprising a three-stranded β-sheet and two α-helices^23^,^24^. Interestingly, the number of POTRA domains can range from one in the mitochondrial Tob55, three in the chloroplast homolog Toc75-V up to seven in BamA from *Myxococcus xanthus*.

Regardless of the number of POTRA domains, a comparative study of 567 such domains identified the most C-terminal domain as the most conserved one^25^. Accordingly, it can be speculated that in the process of organelle evolution the mitochondrial BamA homolog, Tob55, evolved in a way that it retained the most C-terminal POTRA domain as a minimal motif for β-barrel assembly. This assumption is in line with the discovery that POTRA domains 1-4 are dispensable for viability in *Neisseria meningitidis*^26^. This study suggested that multiple POTRA domains are required to facilitate an efficient assembly of larger and more complex substrates^26^. However, in contrast to the situation in *N. meningitidis*, POTRA domains 3-5 are essential in *E. coli*^24^. According to these different requirements of POTRA domains in the various organisms, it is also conceivable that BamA homologs with multiple POTRA domains evolved from a simple ancestral BamA harboring only one such domain.

Very little is known about the exact mechanism by which the individual POTRA domains assist in the assembly of β-barrel proteins. It seems that the POTRA domains facilitate the transfer of the β-barrel substrates from chaperones to the translocase although, at least for mitochondria, a role in the release of the precursor from the TOB complex was also suggested^24^,^27^,^28^. A recent study suggested that the POTRA and the barrel domains of bacterial BamA have to match each other to ensure optimal function of the protein^29^.

In order to better understand the functions of the POTRA domains and the evolutionary conservation between the mitochondrial and the bacterial systems, we constructed hybrid proteins of Tob55 and BamA and expressed them in both eukaryotic and prokaryotic cells. Our findings regarding the functionality of these hybrids indicate that the β-barrel domains of Tob55 and BamA are not interchangeable. Co-expression in yeast cells of these hybrids together with bacterial β-barrel proteins and periplasmic chaperones indicates that the membrane assembly of various β-barrel proteins depends to a different extent on POTRA domains and the chaperones Skp and SurA.

## Results

### Construction and stability of Tob55-BamA hybrid proteins

**Figure 1:**
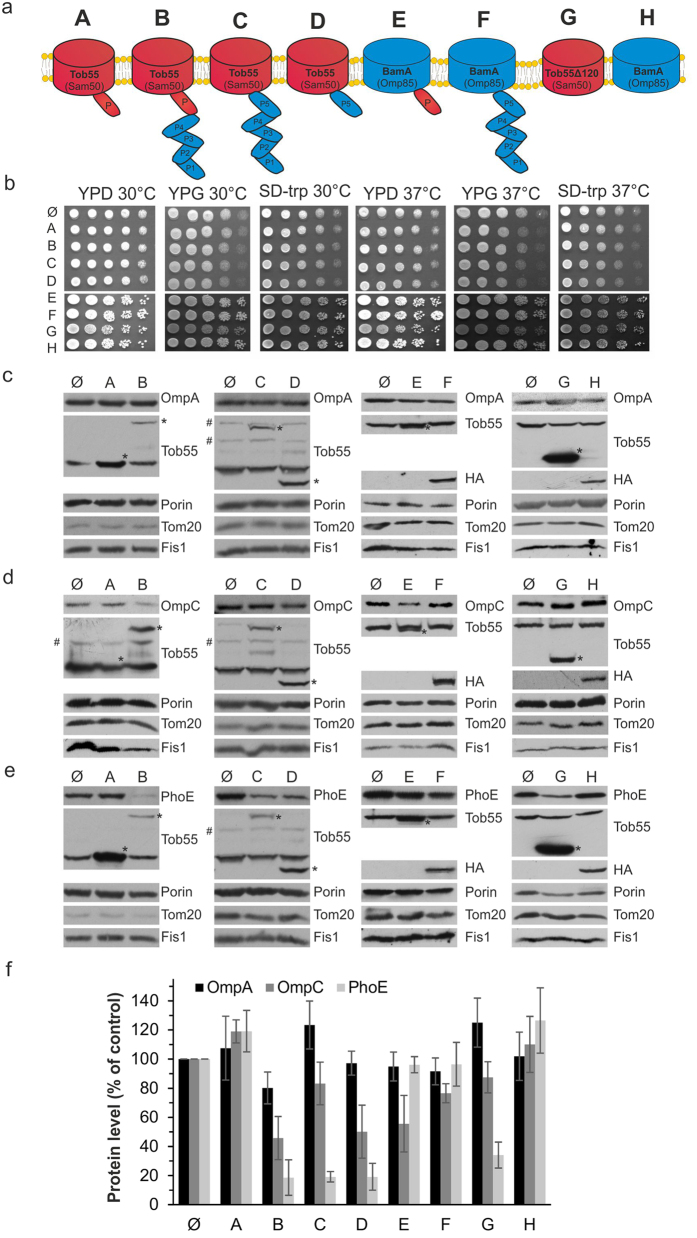
Co-expression of native Tob55 and Tob55-BamA hybrids affects the detected levels of bacterial OMPs. (**a**) Schematic representation of the Tob55/BamA hybrid proteins. Mitochondrial proteins/domains are in red whereas bacterial ones are in blue. (**b**) Yeast cells were transformed with an empty plasmid (Ø) or with a plasmid encoding the indicated hybrid protein. Growth was analyzed by drop-dilution assay at either 30 °C or 37 °C on rich medium containing either glucose (YPD) or glycerol (YPG), or on glucose-containing synthetic medium that lacks tryptophan (SD-Trp). (**c–e**) Crude mitochondria were isolated from cells co-expressing OmpA (**c**), OmpC (**d**) or PhoE (**e**) together with an empty plasmid (Ø) or the indicated hybrid protein. Proteins were analyzed by SDS-PAGE and immunodetection with antibodies against the indicated mitochondrial OM proteins Tob55, Porin (β-barrel protein) as well as Tom20 and Fis1 (single-span proteins). The Tob55/BamA variants are indicated with an asterisk (*) whereas a non-specific band of the Tob55 antibody is depicted with a hash (#). (**f**) The bands of at least three independent experiments as those described in (**c–e**) were quantified and their mean ± s.d. are presented. To allow comparison among the various samples, the levels of Tom20 were taken as internal reference. The intensities of the bands from cells transformed with an empty vector were set to 100%.

To study the ability of various POTRA domains to process β-barrel substrate proteins and to better understand the evolutionary link between the BAM and the TOB machineries, we constructed hybrid Tob55/BamA proteins with different composition of POTRA domains (Fig. 1a). In the first hybrid, POTRA domains 1-4 of *Yersinia enterocolitica* BamA were fused to full-length Tob55 (Fig. 1a, variant B), the second one contains POTRA domains 1-5 of BamA upstream of the membrane-embedded domain of Tob55 (variant C), in the third hybrid the single POTRA domain of Tob55 was replaced by POTRA domain 5 of BamA (variant D). In hybrid E the single POTRA domain of Tob55 was combined with the β-barrel domain of BamA and variants G and H contain only the β-barrel domain of either Tob55 or BamA, respectively (Fig. 1a).

Initially, we aimed to test whether the expression in the same cells of the various hybrid proteins together with the endogenous Tob55 will have any impact on growth of yeast cells. To that goal, plasmids encoding Tob55, BamA or their hybrid proteins were transformed into yeast wild type cells and the effect of these constructs on the growth of the cells was monitored. None of these constructs had any influence on the growth of the cells at normal (30 °C) or elevated (37 °C) temperatures on both fermentable (YPD and SD) and non-fermentable (YPG) carbon source (Fig. 1b). These observations suggest that none of these proteins has a toxic and/or dominant-negative effect on yeast cells.

We previously demonstrated that bacterial β-barrel proteins are targeted to mitochondria upon their expression in yeast cells^15^,^17^,^18^. Thus, we co-expressed in cells harboring endogenous Tob55 the bacterial outer membrane proteins (OMPs) OmpA, OmpC, or PhoE together with the hybrid proteins and tested whether the detected levels of these OMPs are affected by the presence of BamA or the hybrid proteins (Fig. 1c–f). Interestingly, the detected levels of OmpA were hardly affected from the co-expression of BamA or the various hybrid proteins (Fig. 1c,f). In contrast, OmpC was detected in moderately reduced levels in those cells expressing either an hybrid of Tob55 fused to four POTRA domains of BamA or the β-barrel domain of Tob55 fused to a single POTRA domains of BamA (Fig. 1d and f, variants B and D). The amounts of the third OMP, PhoE were heavily reduced in cells expressing hybrids of Tob55 with POTRA domains of BamA as well as the β-barrel domain of Tob55 (Fig. 1d and f, constructs B, C, D and G). Of note, despite this overall reduction in the detected amounts of PhoE, alkaline extraction demonstrated that the detected molecules of the protein were integrated into the membrane even in the presence of the interfering variants (Supplementary Fig. S1). Hence, the reduction in the detected levels of PhoE reflects probably degradation of those molecules that were not integrated into the membrane. Collectively, it appears that the tested bacterial β-barrel proteins are affected to a variable extent by the presence of the hybrid proteins. The interpretation of these variable effects is difficult since the various hybrids proteins are detected in different amounts (Fig. 1c–e). Nevertheless, we assume that the inhibitory effects of the hybrid proteins result from either their ability to bind in a non-productive manner the substrate OMP or from the fact that the hybrid protein is by itself a substrate of the TOB complex and might compete with the biogenesis of other β-barrel proteins. Of note, the levels of the mitochondrial β-barrel proteins Porin and the endogenous Tob55 were not affected from the co-expression of the hybrid proteins (Fig. 1c–e), probably because they are homogeneous robust substrates that suffer less from interferences and sub-optimal conditions.

The expression of constructs A-E and G could be verified by immunodecoration with antibodies against either the full-length Tob55 or its POTRA domain. To detect constructs F and H, an N-terminal HA-tag was added to the proteins and they were immunodetected with anti-HA antibody (Fig. 1c–e). We noted that the detected levels of the hybrids of Tob55 with four or five of the BamA POTRA domains (hybrids B or C, respectively) are rather low (Fig. 1c–e), suggesting that these hybrids are less stable.

### Rescue of Tob55 depletion by Tob55 POTRA mutants

**Figure 2:**
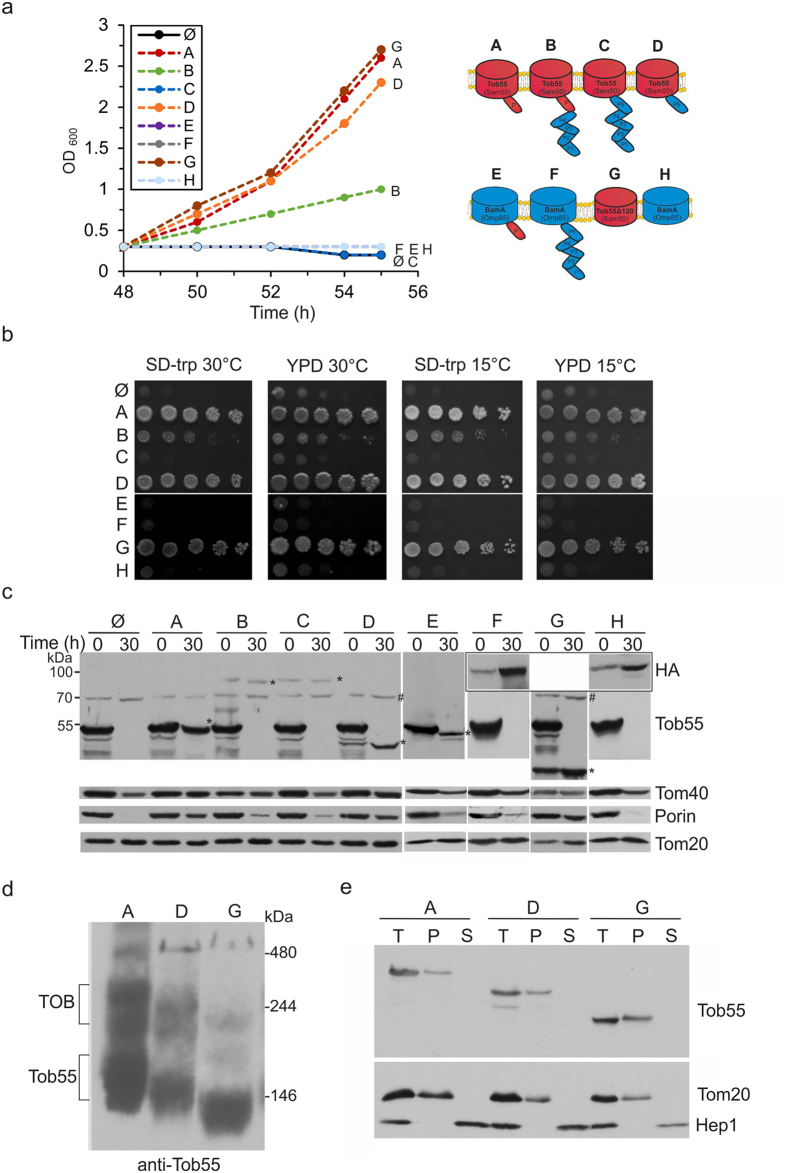
BamA and Tob55 are not interchangeable. (**a**) Plasmids encoding Tob55, BamA, or the various Tob55/BamA hybrids were transformed into a strain expressing Tob55 under the control of an inducible *GAL10* promoter. Cells were grown first on galactose and then were shifted to glucose-containing medium. After 48 h on glucose, all cells were diluted to the same OD_600_ and their growth was further monitored at the indicated time periods. (**b**) The cells described in (**a**) were grown initially for 30 h on galactose-containing medium and then were analyzed at the indicated temperatures by drop-dilution assay on synthetic (SD-Trp) or rich (YPD) glucose-containing medium. (**c**) Cells as in (**a**) were grown first on galactose-containing medium and then were shifted to glucose-containing medium. Cells were harvested directly before the shift (t = 0) or 30 h afterwards (t = 30 h). Proteins were analysed by SDS-PAGE and immunodetection with antibodies against the indicated proteins or the HA-tag. The Tob55-BamA variants are indicated with an asterisk (*) whereas a non-specific band of the Tob55 antibody is depicted with a hash (#). (**d**) Mitochondria were isolated from the Tob55-depleted cells expressing plasmid-encoded Tob55 (variant A), hybrid D, or variant G. The organelles were solubilized with 1% digitonin and were analysed by BN-PAGE and immunodecoration with antibody against Tob55. The bands corresponding to the TOB complex and Tob55 monomeric forms are indicated. (**e**) Mitochondria (Total, T) described in part (**d**) were subjected to alkaline extraction separating membrane-embedded proteins in the pellet (P) from soluble proteins in the supernatant (S). Proteins were analysed by SDS-PAGE and immunodecoration with antibodies against Tob55, Tom20, or Hep1 (a soluble protein in the mitochondrial matrix). For clarity, a letter code of the relevant variants and their scheme are shown.

Previous studies in bacteria suggested that BamA orthologues function in a species-specific manner^21^,^29^,^30^. We thus asked whether the mitochondrial and the bacterial homologues are interchangeable. To that goal we employed a strain where the essential *TOB55* gene is under the regulation of the inducible *GAL10* promoter^11^,^31^. Growing this strain in galactose-containing medium causes up-regulation of TOB55 expression whereas growth on medium with glucose results in depletion of Tob55. Since *TOB55* is an essential gene, growth of this strain on glucose will stop after certain time once Tob55 will be depleted to such low levels, which cannot support viability anymore. We transformed this strain with plasmids encoding the various Tob55/BamA hybrids and asked which one of those can rescue the growth phenotype of Tob55 depletion. As expected, an empty vector could not rescue the phenotype both in liquid and on solid medium while plasmid-encoded TOB55 assured viability (Fig. 2a and b).

Interestingly, variants that harbor the β-barrel domain of Tob55 alone or in combination with POTRA5 of BamA (hybrids G and D, respectively) could completely rescue the absence of native Tob55 whereas a hybrid combined of the Tob55 fused to POTRA domains 1-4 (hybrid B) could do so only partially. The full functionality of the Tob55 membrane-embedded domain is in line with previous report^32^. Of note, hybrid C that contains the β-barrel domain of Tob55 fused to the five POTRA domains of BamA did not have any rescue capacity suggesting that the bacterial POTRA domains interfere with stability of the protein and/or functionality. All hybrids containing the β-barrel domain of BamA (E, F and H) were non-functional in these assays (Fig. 2a,b). The complementation capacity of the various constructs is summarized in Table 1. These results are in accordance with recent reports suggesting that BamA requires the accessory proteins BamB-E in order to efficiently assemble substrate proteins into the membrane^7^,^33^. Since these accessory proteins are lipoproteins that require specific lipid modification, we did not aim to introduce them into the mitochondrial system.

To verify that the growth of the Tob55-depleted cells is not caused by residual levels of Tob55, we isolated cells before and 30 h after the shift to glucose-containing medium and analyzed proteins by immunodecoration. Of note, the expression of all Tob55/BamA hybrids could be verified by immunodetection with antibodies against either Tob55 (constructs A-E and G) or the N-terminal HA-tag (constructs F and H, Fig. 2c). As expected, endogenous Tob55 was detected in high levels upon growth on galactose but completely disappeared after 30 hr on glucose. Concomitantly, the levels of the mitochondrial β-barrels proteins Tom40 and Porin, but not the helical protein Tom20, were also reduced after 30 h in those strains expressing non-functional Tob55/BamA hybrids (Fig. 2c, compare 0 to 30 h). Tob55 itself and its functional variants (D and G) were detected in higher levels than variant B, which has only a partial complementation capacity, and even more so compared to the non-functional hybrid C (Fig. 2c). Hence, it appears that the stability of the variants is correlated to their rescue capacity. However, it is hard to conclude whether the inability of hybrids B and C to efficiently complement the absence of Tob55 results from compromised functionality, insufficient levels, or both.

Next we asked whether the rescuing variants were assembled into the TOB complex. To address this point we isolated mitochondria from the rescued cells and analyzed them by blue native (BN)-PAGE. As anticipated, we observed a smaller TOB complex in those organelles containing only the β-barrel domain of Tob55 (variant G) or this domain together with BamA POTRA5 (variant D), which is smaller than the Tob55 POTRA domain (Fig. 2d). Since the rescuing constructs are over-expressed as compared to the other TOB subunits Tob38 and Mas37, monomeric unassembled forms of all three constructs (A, D, and G) were also detected (Fig. 2d). Furthermore, similar to native Tob55, variants B, D and G could not be extracted from the membrane by alkaline solution suggesting that they are embedded in the membrane (Fig. 2e and Supplementary Fig. S2). These findings suggest that the POTRA domain is neither required for the proper assembly of Tob55 into the TOB complex nor for the membrane integration of the protein.

In contrast to the behavior of the functional hybrids, alkaline extraction assay demonstrated that the non-functional variants containing four bacterial POTRA domains or the β-barrel domain of BamA (C, E, F, and H) were only partially integrated into the membrane (Supplementary Fig. S2). Along this line, variants F and H were detected only within low molecular weight species by BN-PAGE suggesting that they could not assemble into the TOB complex (Supplementary Fig. S2). Unfortunately, constructs B, C and E could not be detected by BN-PAGE and immunodecoration (data not shown), probably due to their low expression levels and/or inaccessibility of epitopes under native conditions. The various properties of the different variants are reviewed in Table 1. Taken together, these observations suggest a correlation between the efficiency of the membrane integration of a certain variant, its stability and its capacity to support function.

### Tob55-BamA variants cannot functionally replace BamA

**Figure 3:**
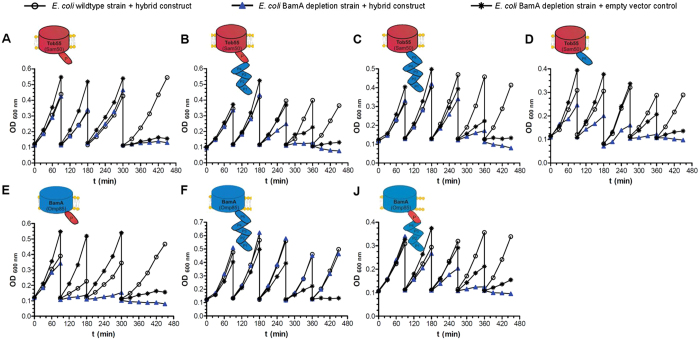
Tob55 cannot rescue *E. coli* cells depleted for BamA. *E. coli* wildtype cells harboring the plasmid for AHTC-inducible expression of the indicated constructs (open circles), and *E. coli* BamA-depleted strain grown under non-permissive conditions harboring either a plasmid for inducible expression of the indicated constructs (filled triangles) or an empty vector as control (asterisks) were grown in liquid culture and the OD_600_ was monitored over time. Sharp drops in the OD_600_ of the various strains indicate the time points when the growing cells were diluted into fresh medium. A letter code of the relevant variant and its scheme are shown above each experiment.

We next asked whether the Tob55-BamA variants can complement the absence of BamA. Since BamA is essential for viability of *E. coli* cells, the functionality of Tob55 and the Tob55-BamA hybrids was tested in cells depleted for BamA^34^. For complementation in *E. coli*, we constructed an additional hybrid in which POTRA5 of BamA, which is the most conserved one, was exchanged by the eukaryotic POTRA domain of Tob55 (Fig. 3, hybrid J). We observed that neither Tob55 nor any of the Tob55-BamA hybrids was able to rescue growth of *E. coli* cells (Fig. 3 and Table 1). As a positive control, we observed that a strain bearing plasmid-encoded native BamA had a growth rate comparable to that of a wild type strain (Fig. 3, construct F). Considering this lack of complementation by full-length Tob55 and the previous knowledge that the bacterial POTRA5 domain is crucial for viability of bacterial cells, POTRA-less variants of both Tob55 and BamA (Fig. 1, constructs G and H, respectively) were not included in this experiment. To exclude the possibility that one or more of the constructs has a toxic effect, all the tested constructs were expressed also in wild type cells harboring normal levels of endogenous BamA. In all of these samples (expressing constructs A-F or J) cells were able to grow at least for the indicated time frame suggesting that none of these variants is harmful to *E. coli* cells (Fig. 3).

To assess if Tob55 and the Tob55/BamA hybrid proteins were indeed expressed and inserted into the OM in *E. coli* cells, expression of the plasmid-encoded proteins was induced under conditions of BamA-depletion. Then we isolated membranes, performed urea extraction and analyzed the samples by Western blots using antibodies directed against Tob55 or BamA (Supplementary Fig. S3). As a control for the behavior of *bona fide* OM proteins, we analyzed also OmpC that fractionated in all assays preferentially with the pellet fraction. Our data show that Tob55 and all hybrid proteins were expressed (Supplementary Fig. S3). However, a notable fraction of Tob55 and its hybrids was extracted upon the urea treatment and was found in the supernatant. Only the hybrid protein D (Tob55 β-barrel fused to the BamA POTRA5) was found exclusively in the pellet fraction. Whereas the endogenous BamA (WT) was detected exclusively in the pellet fraction, constructs C, F and J were detected also in the supernatant (Supplementary Fig. S3). This indicates that these three constructs can in principle, be inserted into the OM, however, most likely due to their overexpression a certain portion is not integrated properly into the OM. Collectively, these results indicate that the lack of functional complementation of the absence of BamA by Tob55 and the Tob55/BamA hybrids is not due to lack of expression. We are unable to conclude whether this lack of functionality is due to differences between Tob55 and BamA in the behavior of the β-barrel domain or results from the inability of the hybrid proteins to interact via their POTRA domain(s) with other Bam subunits, like for example, the essential component BamD.

### Variable dependence of assembly of β-barrel proteins on POTRA domains

We previously demonstrated that bacterial OMPs can be integrated into the mitochondrial OM upon their expression in yeast cells and that this assembly depends on the TOB complex^15^. As the viability of bacteria requires at least one POTRA domain while Tob55 is functional also without this domain, we next aimed to investigate how such POTRA-less Tob55 or variants with bacterial POTRA-domains can deal with bacterial OM proteins expressed in yeast cells. To that goal, we expressed bacterial OmpA, OmpC or PhoE in cells harboring native Tob55, POTRA-less Tob55 (construct G) or the two functional hybrids B and D. The *Y. enterocolitica* BamA can functionally replace the *E. coli* BamA (data not shown), and thus its POTRA domains can correctly recognize *E. coli* substrate proteins.

**Figure 4:**
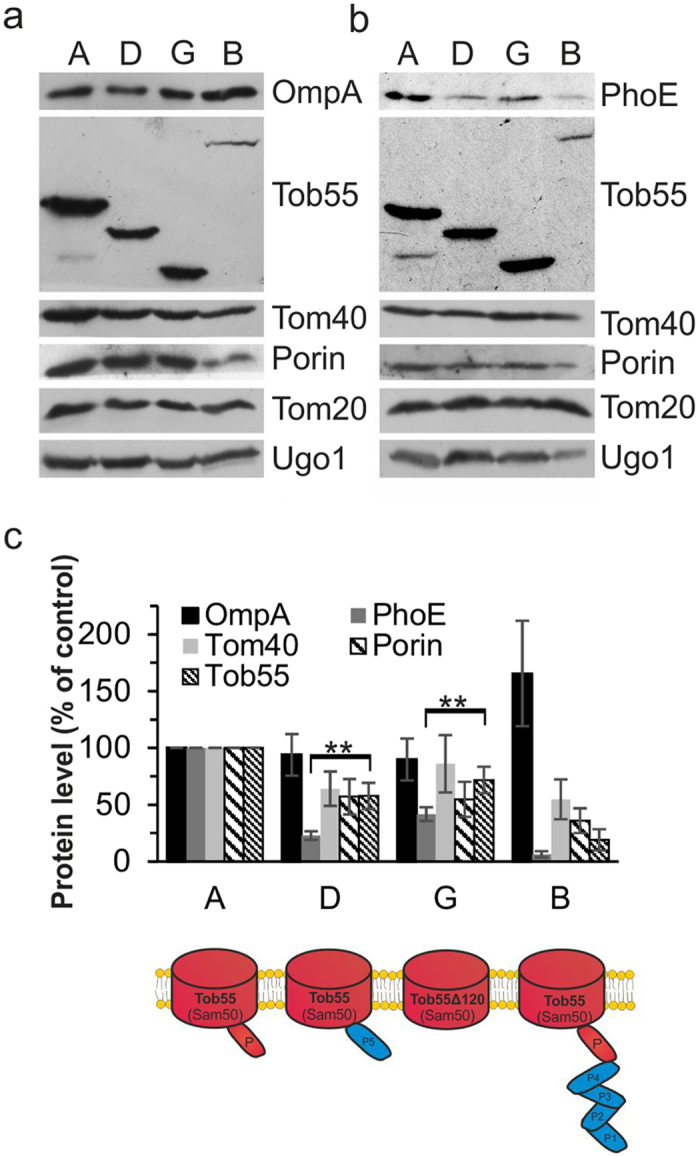
Cells harboring hybrid Tob55/BamA proteins contain lower levels of PhoE. (**a**) Crude mitochondria were isolated from Tob55-depleted cells co-expressing OmpA and either plasmid-encoded Tob55 or the indicated hybrid protein. Proteins were analyzed by SDS-PAGE and immunodetection with antibodies against OmpA, and the indicated mitochondrial proteins. (**b**) Crude mitochondria were isolated from Tob55-depleted cells co-expressing PhoE and the indicated hybrid protein. Further treatment and analysis were as described in part (**a**). (**c**) The bands of at least three experiments as those described in (**a**) and (**b**) were quantified and their means ± s.d. are presented. To allow comparison among the various samples, the levels of Tom20 were taken as internal reference. The intensities of the bands from cells transformed with native Tob55 (construct A) were set to 100%. **p value < 0.01. A schematic representation of the relevant Tob55 variants is included.

Interestingly, OmpA was detected in similar levels irrespective of the presence or composition of the POTRA domains (Fig. 4a and c). The levels of the mitochondrial β-barrel proteins Tom40 and Porin, as well as the bacterial protein OmpC, were only moderately decreased in cells with Tob55 variants (Fig. 4a–c and Supplementary Fig. S4). In contrast, the chloroplast β-barrel protein Oep37, which can be assembled into the mitochondrial OM upon its expression in yeast cells^31^, was significantly affected by the presence of hybrid D (Supplementary Fig. S4). Similarly, PhoE was detected in dramatically reduced levels in cells harboring the non-native variants (Fig. 4b,c). Of note, despite this reduction in the total PhoE levels, the detected molecules were integrated into the mitochondrial OM as monitored by alkaline extraction (Supplementary Fig. S5). We assume that this observation reflects degradation of PhoE molecules that were not assembled into the membrane. As expected, the amounts of the helical OM proteins Tom20 and Ugo1 were not influenced by the presence of the various variants (Fig. 4a,b), indicating that the aforementioned effects are specific for β-barrel proteins.

Of note, the levels of the Tob55 variants D and G were moderately lower than the level of the native protein whereas those of variant B were dramatically reduced. Nevertheless, the relative reduction in the levels of Oep37 and PhoE in these cells was significantly larger than the decrease in the amounts of the Tob55 variants (Fig. 4c, constructs D and G and Fig. S4 construct D). These findings demonstrate that the membrane assembly of different β-barrel proteins depends to a variable extent on the presence of the POTRA domains. Among the tested proteins, the efficiency of membrane integration of PhoE is especially dependent on the correct matching between the POTRA and the barrel domains as it is inhibited in hybrids containing the mitochondrial β-barrel moiety and bacterial POTRA domain(s).

### Membrane integration of PhoE is enhanced by the periplasmic chaperone Skp

We have recently reported that co-expression of the periplasmic chaperone Skp, but not of SurA, in the mitochondrial IMS can enhance the stability of the bacterial trimeric autotransporter YadA when the latter is expressed in yeast cells and gets assembled into the mitochondrial OM^18^. We next wanted to test whether this influence can be observed also for other bacterial OMPs and how the chaperone’s contribution is dependent on the presence of POTRA domains. To that aim we employed cells depleted for endogenous Tob55 and complemented with plasmid-encoded Tob55, POTRA-less Tob55, or the latter fused to bacterial POTRA5.

**Figure 5:**
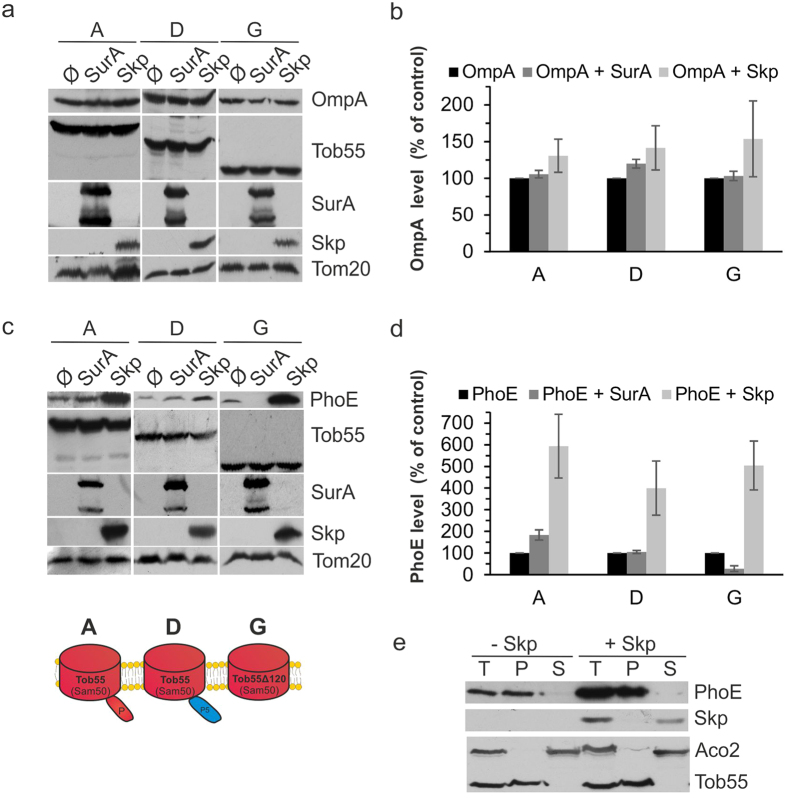
PhoE and OmpA have variable dependencies on chaperones. (**a**) Crude mitochondria were isolated from Tob55-depleted cells co-expressing OmpA and either plasmid-encoded Tob55 or the indicated variants. In addition, these cells were transformed with an empty plasmid (Ø) or a plasmid encoding mitochondrially-targeted SurA or Skp. Proteins were analyzed by SDS-PAGE and immunodetection with the indicated antibodies. (**b**) The bands of at least three independent experiments as those described in (**a**) were quantified and their means ± s.d. are presented. To allow comparison among the various samples, the levels of Tom20 were taken as internal reference. The intensities of the bands from cells transformed with native Tob55 (construct A) in the absence of chaperone were set to 100%. (**c,d**) Crude mitochondria were isolated from Tob55-depleted cells co-expressing PhoE, the indicated Tob55 variant, and either SurA or Skp. Further treatment and analysis were as described in parts (**a**) and (**b**). (**e**) Mitochondria (Total, T) were isolated from cells co-expressing plasmid-encoded native Tob55 (construct A) and PhoE in the presence or absence of Skp. The organelles were subjected to alkaline extraction separating membrane-embedded proteins in the pellet (P) from soluble proteins in the supernatant (S). Proteins were analyzed by SDS-PAGE and immunodecoration with antibodies against PhoE, Skp, Aco2 (a soluble protein in the mitochondrial matrix), and Tob55. A schematic representation of the relevant Tob55 variants is included.

OmpA or PhoE were co-expressed with either Skp or SurA in these cells. Of note, both bacterial chaperones are targeted to the mitochondrial IMS in these cells^18^. The levels of OmpA were not significantly altered by the presence of Skp or SurA (Fig. 5a,b). The apparent independency of OmpA levels of the presence of periplasmic chaperones or the various Tob55/BamA hybrids raises the theoretical possibility that the membrane assembly of OmpA can occur without the involvement of the mitochondrial import system. To test this possibility we monitored the levels of OmpA in a strain where the expression of Tob55 is controlled by the inducible *GAL10* promoter. Similarly to other mitochondrial β-barrel proteins like Porin and Tom40, the detected levels of OmpA were dramatically reduced upon depletion of Tob55 (Supplementary Fig. S6), suggesting that OmpA requires the TOB complex for its biogenesis.

In contrast to the behavior of OmpA, the co-expression of Skp dramatically enhanced the levels of PhoE in all tested cells. Interestingly, the presence of SurA moderately increased the levels of PhoE in cells harboring native Tob55 but resulted in decreased PhoE amounts in cells containing the POTRA-less Tob55 variant (Fig. 5c,d).

The elevated levels of PhoE upon co-expression with Skp raised the question whether the latter simply stabilizes more substrate molecules in the mitochondrial IMS or can also enhance their membrane integration. To address this question we performed alkaline extraction of mitochondria harboring PhoE alone or in combination with Skp. We observed that the elevated amounts of PhoE reflect membrane-embedded molecules (Fig. 5e), suggesting that the activity of Skp indeed improves the membrane integration competence of the substrate.

Collectively, these findings indicate that OmpA requires neither POTRA domains nor the assistance of soluble bacterial chaperones for optimal membrane integration in mitochondria whereas PhoE can be supported by Skp. The negative effect of SurA in combination with a POTRA-less variant might be explained by the requirement for a POTRA domain to induce a release of substrate from SurA. In the absence of such a domain, SurA can bind a substrate protein but does not release it for further assembly into the membrane and hence altogether has a rather inhibiting effect. This assumption is supported by a recent report describing a ternary complex formed by BamA (mainly via POTRA domain 2), SurA, and β-barrel nascent protein^35^. Our observations are also in line with previous works suggesting that the individual or combinatorial deletion of periplasmic chaperones or non-essential Bam components differentially affect the assembly of various groups of β-barrel proteins, proposing that there are classes of substrates with variable dependencies for efficient assembly^36^,^37^,^38^.

### OmpX and 2xOmpX have similar requirements for chaperones and POTRA domain

Our results so far demonstrated a clear difference between OmpA and PhoE regarding their dependency on POTRA domains and soluble chaperones. A possible explanation for this variability can be their different size. The β-barrel domain of OmpA is built from 8 β-strands whereas that of PhoE from 16 β-strands. Alternatively, their different behavior can be related to variability in parameters like overall charge, hydrophobicity, kinetics of folding, or others. To directly test this issue, we used the bacterial protein OmpX (8 β-strands) and its duplicated construct 2xOmpX (16 β-strands). The duplicated protein was reported to insert correctly into membranes and to form a pore with double the size of the normal OmpX^39^. Assuming that both constructs share the same chemiophysical characteristics, the major difference between them is the size.

**Figure 6:**
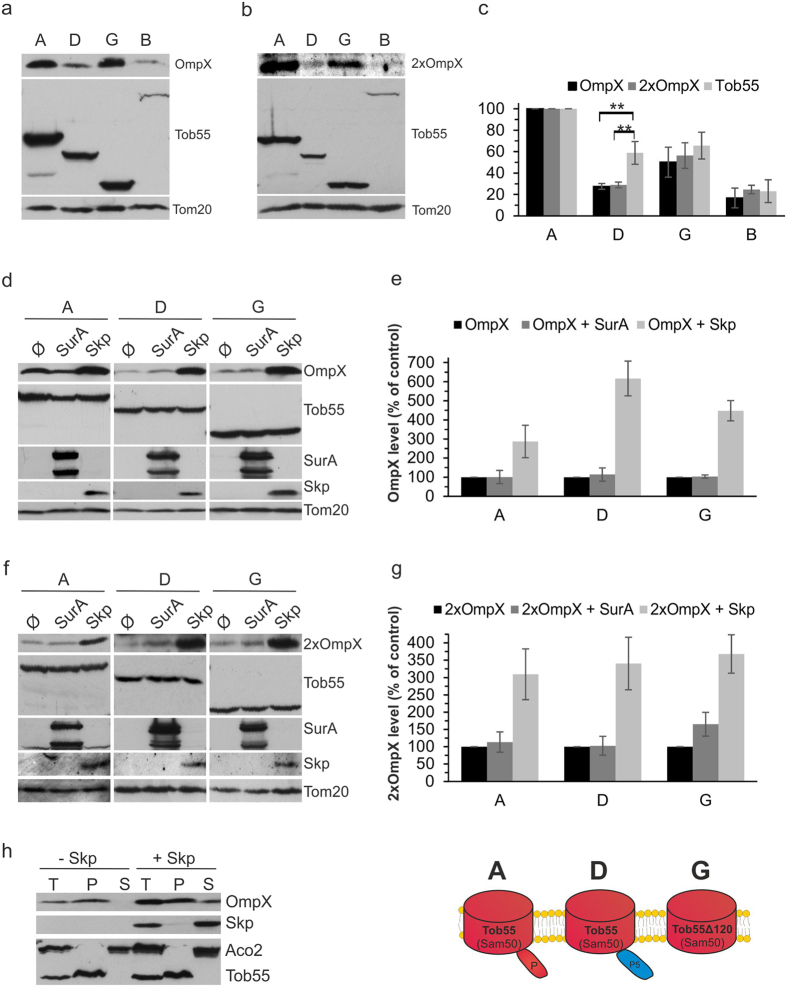
OmpX and 2xOmpX are similarly dependent on chaperones. (**a,b**) Crude mitochondria were isolated from Tob55-depleted cells co-expressing OmpX (**a**) or 2xOmpX (**b**) and either plasmid-encoded Tob55 or the indicated hybrid protein. Proteins were analyzed by SDS-PAGE and immunodetection with the indicated antibodies. (**c**) The bands of at least three experiments as those described in (**a**) and (**b**) were quantified and their means ± s.d. are presented. To allow comparison among the various samples, the levels of Tom20 were taken as internal reference. The intensities of the bands from cells transformed with native Tob55 (construct A) were set to 100%. **p value < 0.01. (**d,e**) Crude mitochondria were isolated from Tob55-depleted cells co-expressing OmpX, the indicated Tob55 variant, and either SurA or Skp. Further treatment and analysis were as described in the legends to Fig. 5 parts (**a**) and (**b**). (**f,g**) Crude mitochondria were isolated from Tob55-depleted cells co-expressing 2xOmpX, the indicated Tob55 variant, and either SurA or Skp. Further treatment and analysis were as described in the legends to Fig. 5 parts (**a**) and (**b**). (**h**) Mitochondria (Total, T) were isolated from cells co-expressing plasmid-encoded native Tob55 (construct A) and OmpX in the presence or absence of Skp. The organelles were subjected to alkaline extraction separating membrane-embedded proteins in the pellet (P) from soluble proteins in the supernatant (S). Proteins were analyzed by SDS-PAGE and immunodecoration with the indicated antibodies. A schematic representation of the relevant Tob55 variants is included.

Next, we expressed both proteins in cells depleted for endogenous Tob55 expressing plasmid-encoded Tob55 or its functional variants. Both proteins could be detected in the crude mitochondrial fraction and their detected levels were dramatically decreased in cells harboring Tob55 with bacterial POTRA1-4 (variant B) or POTRA-less Tob55 fused to bacterial POTRA5 (variant D) (Fig. 6a–c). Since in the latter case this reduction was significantly higher than the decrease in the amounts of the Tob55 variant itself, it can be concluded that both versions of OmpX require optimal matching of the POTRA domain(s) of Tob55 with its membrane embedded domain.

Next, we co-expressed the chaperones Skp or SurA in these cells. Interestingly, the levels of both OmpX variants were highly increased by the presence of Skp whereas SurA had hardly any influence (Fig. 6d–g). Intriguingly, the presence of Skp not only enhanced the overall levels of detected OmpX but also of the membrane integrated portion of the protein. Nevertheless, some portion of OmpX molecules was found also in the soluble fraction, raising the possibility that they were not import competent (Fig. 6h). Of note, Skp-dependent augmentation was observed also in cells expressing the POTRA-less Tob55 (construct G), supporting the notion that Skp can function also in the absence of POTRA domains. Generally, the similar behavior of OmpX and its duplicate (2xOmpX) argues against size by itself as a major criterion for variable dependencies of substrate β-barrel proteins.

## Discussion

The central components of the machineries that integrate β-barrel proteins into membranes, BamA and Tob55, are conserved from bacteria to human^1^,^14^. Indeed, both complexes are able to deal with substrate proteins from the respective other system and to manage their membrane insertion^15^,^16^,^40^. However, an open question was whether the bacterial BamA and the mitochondrial Tob55 are functionally interchangeable. The current work demonstrates that these proteins cannot replace each other. Hence, although they fulfill similar function in their corresponding membranes, it appears that the need of each one of them to co-function with specific auxiliary proteins (BamB-E in bacteria and Mas37 and Tob38 in mitochondria) does not allow such a replacement. Furthermore, the sub-optimal matching between the β-barrel domains of BamA and Tob55 and the foreign membrane might provide an additional explanation for the lack of function in the other system.

Structural studies suggested that destabilization of the membrane environment around the β-barrel domain of BamA/Tob55 might be important for membrane integration of substrate proteins^21^. However, it can be assumed that BamA and Tob55 are optimized to function within their corresponding membranes, which vary significantly from each other in their lipid composition. Thus, one can speculate that both proteins were fine-tuned to disturb their residence membranes but might not be able to do so in a foreign membrane. The observed lack of functional replacement is also in line with previous reports that even within the prokaryotes kingdom BamA appears to function in a species-specific manner and *E. coli* BamA can be substituted only by BamA homologues from closely related species^21^,^29^,^41^.

In addition to BamA, we investigated also the ability of hybrid Tob55/BamA to functionally replace either Tob55 or BamA. Interestingly, POTRA-less Tob55 or Tob55 where the eukaryotic POTRA domain was replaced by bacterial POTRA5 were functional. The addition of POTRA1-4 to Tob55 resulted in an unstable protein that was expressed at lower levels and had only a weak complementation capacity. These findings support the notion that although several functions were suggested for the single eukaryotic POTRA domain^27^,^28^, it is not absolutely required for function^32^. The inability of all hybrids to replace BamA in bacteria are in line with a recent report suggesting that the bacterial POTRA and the barrel domains should be optimally tailored in order to obtain a functional protein^29^.

To better understand the contribution of the POTRA domains to the overall biogenesis of β-barrel proteins from both prokaryotic and eukaryotic origin, we used mitochondria of yeast cells. This system can deal with various bacterial OM proteins and allows also the evaluation of the contribution of the periplasmic chaperones SurA and Skp when these are targeted to the mitochondrial intermembrane space^15^,^18^, which is the topological equivalent of the bacterial periplasm. Using this approach we observed clear differences in the dependency of the various proteins on the presence of POTRA domain(s). Whereas OmpA was integrated into the membrane independently of the presence of POTRA domains, mitochondrial β-barrel proteins and OmpX were moderately affected, and the levels of PhoE were dramatically reduced. Hence, it seems that substrate proteins are dependent to a different extent on POTRA domains. Since both OmpA and OmpX are monomeric proteins harboring 8 β-strands and OmpX is even smaller than OmpA, it appears that this variable dependency is not related only to the size of the proteins.

Similar variability was observed also regarding the dependency on the amount of Tob55 or its variants. Cells that had lower levels of the B variant of Tob55 grew slower than control cells and had reduced levels of mitochondrial β-barrel proteins or the bacterial proteins PhoE and OmpX. In sharp contrast, OmpA was detected in these cells in slightly elevated amounts. These findings complement very recent observations where lower levels of Bam components resulted in differential effect on the levels of various OM proteins^38^. Of note, Mahoney *et al*. reported that mutant cells that harbor lower levels of BamA or BamD contained also reduced level of some OM proteins whereas others like OmpA were not affected. Hence, these similar observations between mitochondria and bacteria stress again the conservation in the basic principles of membrane assembly between these two systems.

Finally, the current study sheds new light on the differential role of the periplasmic chaperones SurA and Skp. Studies in *E. coli* suggest that SurA is the primary chaperone but most substrates can efficiently use Skp when SurA is absent^42^. Only certain OM proteins, like LptD, specifically require SurA and it might be that structural features determine which OMPs require SurA for their assembly^36^. However, it should be noted that in *N. meningitidis* the situation appears to be different and Skp is the more important chaperone^4^.

In the mitochondrial system we observed that SurA plays only a very minor role, if at all, and in one case had even an inhibitory effect. Skp, in contrast can increase, upon its expression in yeast cells, the levels of PhoE and OmpX but not of OmpA or the mitochondrial β-barrel proteins. The similar behavior of OmpX and its duplicate 2xOmpX substantiates the notion that chemiophysical characteristics rather than size dictates the dependency on POTRA domains and periplasmic chaperones. We propose that the different effects of Skp and SurA might be related to the different mode of action of both chaperones. Skp interacts with unfolded substrate proteins and thus hinders non-productive aggregation or degradation^43^. This activity is observed also *in vitro* and does not seem to be dependent on other proteins^44^. In contrast, SurA was suggested to interact with POTRA domains of BamA^45^, and it was proposed that secretion of the passenger domain of autotransporter proteins might involve a stepwise transfer of polypeptide segments from SurA to the POTRA domains^46^. Supporting this notion is a recent report describing the detection of a ternary complex formed by BamA (via POTRA domain 2), SurA, and β-barrel substrate protein^35^. Hence, we hypothesize that in the mitochondrial system SurA might still bind its substrate proteins. However, since the TOB complex lacks bacterial POTRA domains, such a binding is not productive because SurA cannot efficiently transfer these substrates to the insertion machinery. Skp, in contrast, can stabilize its substrate independently of the presence of bacterial effectors and thus its positive effect is observed even in the mitochondrial system. This proposal is further supported by our previous observations that Skp, but not SurA, can stabilize the bacterial trimeric autotransporter YadA when the latter was on its way to become assembled into the OM of yeast mitochondria^18^.

The more pronounced effect of Skp in comparison to SurA in our experimental system seems to contradict results obtained with *E. coli* cells^3^,^42^. However, our findings might be explained by the partial accumulation of substrate proteins in the IMS, a situation similar to envelope stress in the bacterial periplasm. Upon periplasmic envelope stress, Skp was reported to gain importance, whereas SurA seems to be mainly involved in protein biogenesis under more physiological conditions^3^,^42^. Another explanation for the apparent importance of SurA in *E. coli* cells is its significance for the proper biogenesis of LptD in this organism. The absence of SurA reduces the levels of LptD and hence indirectly affects the lipid composition of the OM, which in turns might cause a decrease in the membrane insertion of β-barrel proteins in SurA deletion strains^3^,^47^.

The current study shed also new light on the robustness of the membrane assembly process of OmpA. This protein was hardly affected by any Tob55 variant and even more strikingly, its levels were constant in cells harboring a Tob55 variant which was detected in approx. 20% of the levels of native Tob55. In sharp contrast to OmpA levels, levels of mitochondrial β-barrel proteins and all other tested bacterial OMPs were clearly reduced under these conditions (presence of construct B). These observations are in line with recent findings of Mahoney *et al*. in *E. coli* cells where in contrast to other bacterial β-barrel proteins, OmpA was not directly affected by strong depletion of BamA and BamD^38^. Altogether, these observations, suggest that OmpA can be efficiently integrated into the membrane even by low levels of a functional insertion complex and without major help from chaperones and POTRA domains.

In summary, this study demonstrates that although mitochondria evolved from bacteria, the evolutionary drift of the translocation machineries for β-barrel proteins makes their central subunits non-interchangeable. Nevertheless, the similarity in the basic membrane assembly process allows the usage of mitochondria to obtain insights into general aspects of biogenesis of these proteins. Employing this system, we observed that bacterial β-barrel proteins depend to a variable extent on the presence of POTRA domains and periplasmic chaperones.

## Methods

### Yeast strains and growth methods

Standard genetic techniques were used for growth and manipulation of yeast strains. The wild-type strain W303α was routinely utilized unless indicated otherwise. YPH499 *GAL10*-*TOB55* strain was employed for Tob55 complementation assays^11^. Yeast strains were grown in standard rich medium YP (2% [w/v] bacto peptone, 1% [w/v] yeast extract), or synthetic medium (0.67% [w/v] bacto-yeast nitrogen base without amino acids with glucose or galactose (2% [w/v]). For drop dilution assays, cells were cultured to an OD_600_ of 1.0 and diluted in five-fold increment followed by spotting 5 μl of each cell suspension on the corresponding solid medium.

### Bacterial strains and growth methods

*Escherichia coli* K-12 derivative MC4100A and the corresponding BamA depletion strain were described before^34^. These strains were transformed with the pASK-IBA2 expression vector encoding wild type BamA or Tob55/BamA hybrid constructs. For overnight cultures, bacterial strains were grown at 37 °C in Luria–Bertani (LB) broth with 100 mg/ml ampicillin. The BamA depletion strain was additionally supplied with 50 mg/ml kanamycin and 0.2% arabinose (w/v) (to allow the endogenous expression of BamA).

### Recombinant DNA techniques

Cloning of the bacterial β-barrel proteins OmpA, OmpC, and PhoE into yeast expression vectors was described before^15^. The plasmids for the expression of the bacterial chaperones in mitochondria were described by Ulrich *et al*.^18^. OmpX and 2xOmpX were cloned via PCR amplification from the plasmids pET3bOmpX8 or pET3bOmpX88, respectively (kind gift from Dr. D. Linke). PCR fragments were ligated into a yeast expression vector, as described for PhoE^15^, using EcoRI and SalI (OmpX) or NcoI and SalI (2xOmpX) restriction sites.

The Tob55/BamA hybrid constructs are schematically depicted in Fig. 1a. Hybrids for expression in yeast cells are based on BamA from *Y. enterocolitica*. Hybrids C and F were sub-cloned, using EcoRI and SacI restriction sites, from the plasmid pASK_Iba2-Tob55-PD(1-5) (lacking the bacterial signal sequence) or pYX113-BamA, respectively. Construct B was cloned from pASK_Iba2-Tob55-PD(1-4) plasmid without the bacterial signal sequence using PCR amplification. Constructs D, G and H were generated via PCR amplification from plasmid pASK_Iba2-Tob55-PD(1-5), pYX132-Tob55 or pYX132-BamA, respectively, by omitting the non-relevant POTRA domains. Construct E was cloned using PCR amplification from pASK_Iba2-Tob55-PD(1-5) and pYX132-Tob55 plasmids followed by fusion of both PCR fragments via overlapping extension PCR. All hybrid constructs were inserted into yeast expression vector pYX132 using EcoRI and SalI restriction sites, if not indicated differently. For immunodetection, a triple HA-tag was inserted into the EcoRI restriction site on the N-terminus of constructs F and H.

For expression in *E. coli* cells, native Tob55, *E. coli* BamA and their hybrid constructs were cloned into pASK-IBA2 expression vector using Gibson Assembly Master Mix containing T5 Exonuclease (10 U μl^−1^), Phusion Polymerase (2 U μl^−1^) and Taq-DNA-Ligase (40 U μl^−1^). The fragments of the bamA or *TOB55* genes were amplified with KOD hot start DNA polymerase using chromosomal DNA of *E. coli* MC4100^34^ or pGem4-*TOB55* (this study) as templates, respectively. All constructs were verified by DNA sequencing. Primers used are available upon request.

### Functional complementation of Tob55

For complementation experiments, a yeast strain (YPH499-*GAL10*pro-Tob55-His8) with Tob55 under inducible *GAL10*-promoter was transformed with yeast expression plasmids (pYX132) encoding the various Tob55-BamA hybrid proteins. Cells were grown first on galactose-containing medium and then, to deplete the native form of Tob55, they were shifted for 30 hr to glucose-containing medium.

### Functional complementation of BamA

*Escherichia coli* K-12 derivative MC4100A and the corresponding BamA depletion strain were transformed with pASK-IBA2 expression vectors encoding wild type BamA or Tob55/BamA hybrid constructs. For overnight cultures, bacterial strains were grown as described above and then the cultures were re-suspended in fresh medium and diluted to an OD_600_ of 0.1. Bacterial cultures were grown for 2 h at 27 °C before anhydrotetracycline (AHTC) was added to a final concentration of 40 ng ml^−1^. If not stated otherwise, bacteria were allowed to express plasmid-encoded BamA or Tob55/BamA hybrids for another 2 h. Afterwards cultures were harvested by centrifugation and washed twice with PBS to get rid of arabinose in the medium. Both strains were diluted again to an OD_600_ of 0.1 and further grown at 27 °C in LB broth with 100 mg/ml ampicillin and AHTC. To deplete endogenous BamA, *E. coli* MC4100∆*bamA* strain was supplemented with 0.2% glucose (w/v). All strains were grown at 27 °C for 7.5 h and OD_600_ was measured every 30 min. After every 90 min cultures were diluted to an OD_600_ of 0.1 and supplemented as described above.

### Preparation of bacterial outer membranes

Preparation of outer membranes was carried out as described previously^48^. Briefly, 50 ml of bacterial culture were harvested, re-suspended in 500 μl of resuspension buffer containing 500 μg lysozyme. After addition of 3.2 ml water, the samples were incubated for 20 min and the resulting protoplasts were lysed in 5 ml of lysis buffer. Released DNA was digested by the addition of 50 μg of DNase I. Outer membranes were pelleted by centrifugation and after three washing steps re-suspended in SDS sample buffer.

### Urea extraction

Bacterial envelopes were pelleted by centrifugation (290,000 × g, 1 h, 4 °C) and pellets were re-suspended in 1 ml of urea solution (100 mM glycine, 6 M urea, 15 mM Tris-HCl, pH 7.4) and incubated in this suspension for 1 h at 37 °C. Urea-treated samples were re-isolated by centrifugation (290,000 × g, 90 min, 25 °C) and re-suspended in SDS sample buffer.

### Blue native gel electrophoresis

Native complexes of membrane proteins were separated by blue native gel electrophoresis^49^. Isolated mitochondria were collected (25,000 × g, 10 min, 4 °C) and solubilized with 1% digitonin or the indicated detergent. For digitonin the samples were incubated in detergent:protein ratio of 6:1 in buffer N (0.1 mM EDTA, 50 mM NaCl, 10% (v/v) glycerol, 20 mM Tris/HCl, pH 7.4) for 20 min on ice. Non-solubilized material was removed by centrifugation (36,000 × g, 10 min, 4 °C). The supernatant was mixed with 10x loading dye and loaded on a blue native gel with a gradient of 6–13% acrylamide.

### Biochemical procedures

Mitochondria were isolated from yeast cells by differential centrifugation as described before^50^. Subcellular fractionation of yeast cells was performed as described previously^15^. For carbonate extraction, mitochondria (75 μg) were re-suspended in 50 μl of 20 mM HEPES (pH 8.0), then 50 μl of sodium carbonate (200 mM) were added and samples were incubated for 1 h on ice followed by centrifugation (100,000 × g, 45 min, 2 °C). The pellet (membrane fraction) was re-suspended in SDS-PAGE sample buffer and proteins from the supernatant were precipitated with trichloroacetic acid (20%), spun down (30,000 × g, 10 min, 2 °C), washed with 1 ml cold acetone, pelleted again (30,000 × g, 10 min, 2 °C), and resuspened in SDS-PAGE sample buffer. Full uncropped versions of all immunoblot images from all figures are included in Supplementary Fig. S7.

## Additional Information

**How to cite this article**: Pfitzner, A.-K. *et al*. Mitochondrial-bacterial hybrids of BamA/Tob55 suggest variable requirements for the membrane integration of β-barrel proteins. *Sci. Rep.* **6**, 39053; doi: 10.1038/srep39053 (2016).

**Publisher's note:** Springer Nature remains neutral with regard to jurisdictional claims in published maps and institutional affiliations.

## Supplementary Material

Supplementary Figures

## Figures and Tables

**Table 1:** Properties of Tob55/BamA hybrids.

Variant	Replacement of Tob55 or BamA		Membrane insertion		Assembly into TOB complex
	Yeast	*E. coli*	Yeast	*E. coli*	
A (Tob55)	yes	no	yes	part.	yes
B	part.	no	yes	part.	nd
C	no	no	part.	part.	nd
D	yes	no	yes	yes	yes
E	no	no	part.	part.	nd
F (BamA)	no	yes	part.	part.	no
G	yes	nt	yes	nt	yes
H	no	nt	part.	nt	no
J	nt	no	nt	part.	nt

nt, construct was not tested in this experiment; part., partially; nd, hybrid protein was not detectable.
