# Supplementary material for: Mitochondrial-bacterial hybrids of BamA/Tob55 suggest variable requirements for the membrane integration of β-barrel proteins
Source: Sci Rep. 2016 Dec 16;6:39053. doi: 10.1038/srep39053 (PMC5159795; doi:10.1038/srep39053)
Supplement: Supplementary Figures [file srep39053-s1.pdf]

Mitochondrial-bacterial hybrids of BamA/Tob55 suggest variable requirements for the membrane integration of β-barrel proteins

Anna-Katharina Pfitzner, Nadja Steblau, Thomas Ulrich, Philipp Oberhettinger, Ingo B. Autenrieth, Monika Schütz, and Doron Rapaport

Supplementary figures

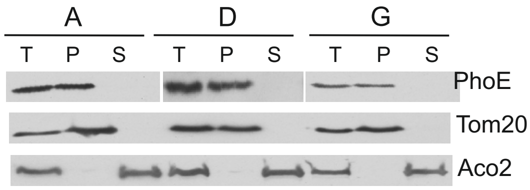

Figure S1: PhoE is membrane inserted in presence of Tob55/BamA variants D and G. Mitochondria from WT cells expressing the indicated Tob55/BamA variant and PhoE were isolated. Mitochondria (Total, T) were subjected to carbonate extraction separating membrane embedded proteins in the pellet (P) from soluble and membrane associated proteins in the supernatant (S). Proteins were analyzed by SDS/PAGE and immunodecoration with antibodies against PhoE, Tom20 (MOM protein) or Aco2 (a soluble protein in the mitochondrial matrix).

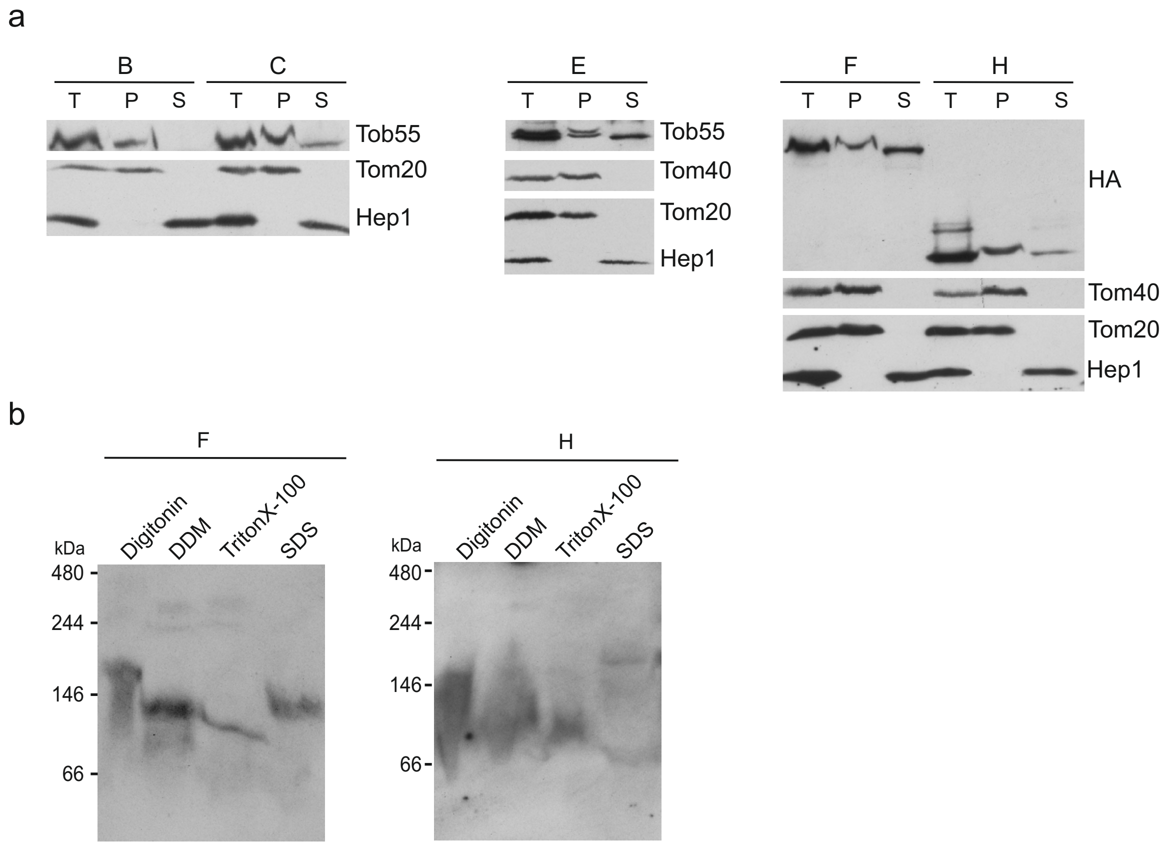

Figure S2: Non-functionalTob55/BamA variants are only partially integrated into the mitochondrial membrane and TOB complex. a. Mitochondria from strains carrying Tob55 under the inducible *GAL10* promoter and expressing the indicated Tob55/BamA variant were isolated 26 hr after shift to glucose-containing medium. Mitochondria (Total, T) were subjected to carbonate extraction separating membrane embedded proteins in the pellet (P) from soluble proteins in the supernatant (S). Proteins were analyzed by SDS-PAGE and immunodecoration with antibodies against the indicated proteins. b. TOB complexes of mitochondria described in (a) were solubilized with the indicated detergent and then analyzed by BN-PAGE and immunodecoration with antibodies against Tob55.

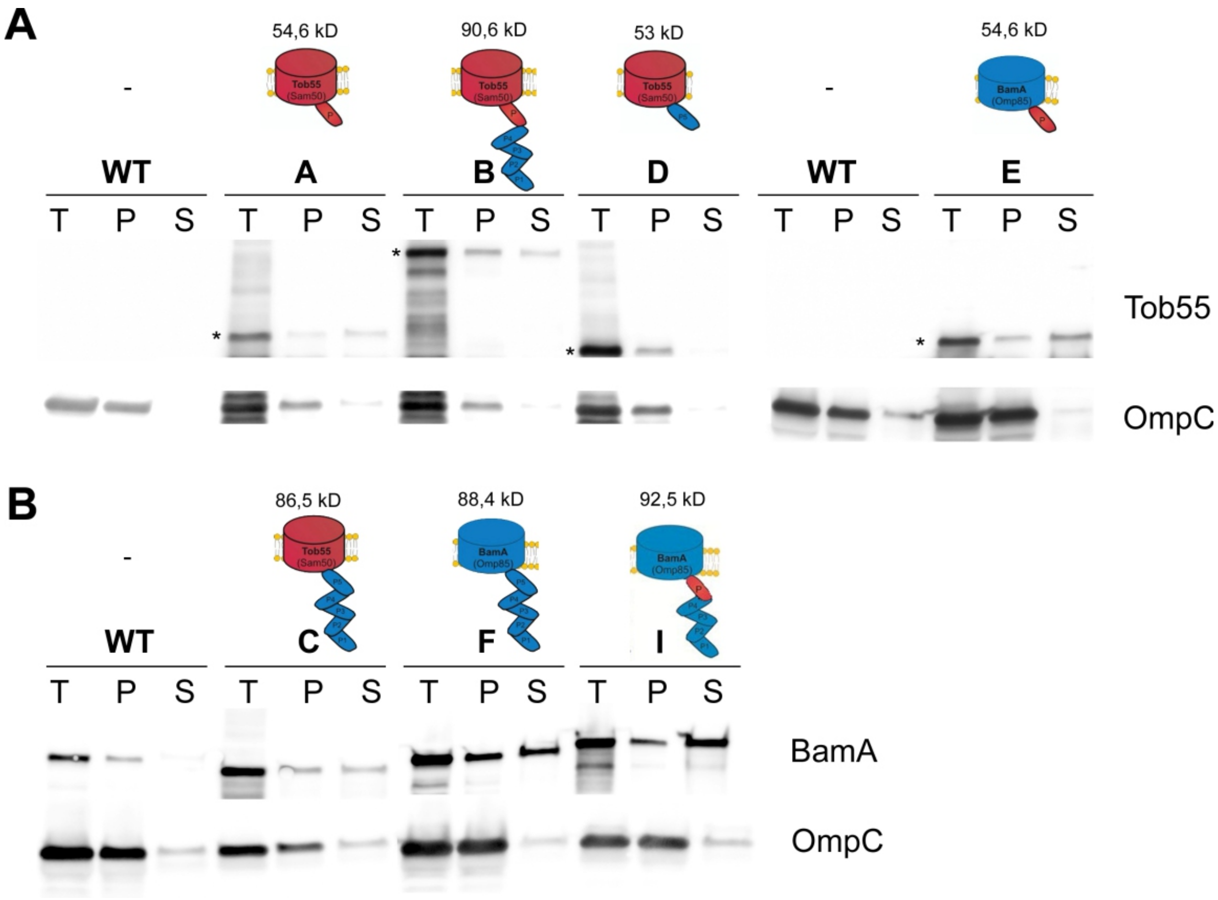

Figure S3: Expression and OM insertion of Tob55/BamA hybrids. The E. coli BamA depletion strain harboring the indicated constructs was grown under non-permissive conditions and a membrane fraction was prepared. An E. coli wild type strain was used as a control for expression and membrane insertion of endogenous BamA. Total membranes (T) were extracted with urea and soluble (S) and insoluble material (P= pellet) were separated by centrifugation and analysed by SDS-PAGE and immunodetection with antibodies directed against (A) Tob55 or (B) BamA. The distribution of the OM protein OmpC was used as a control. The composition of the hybrid proteins is illustrated with small cartoons and their calculated molecular weight is indicated above the cartoons.

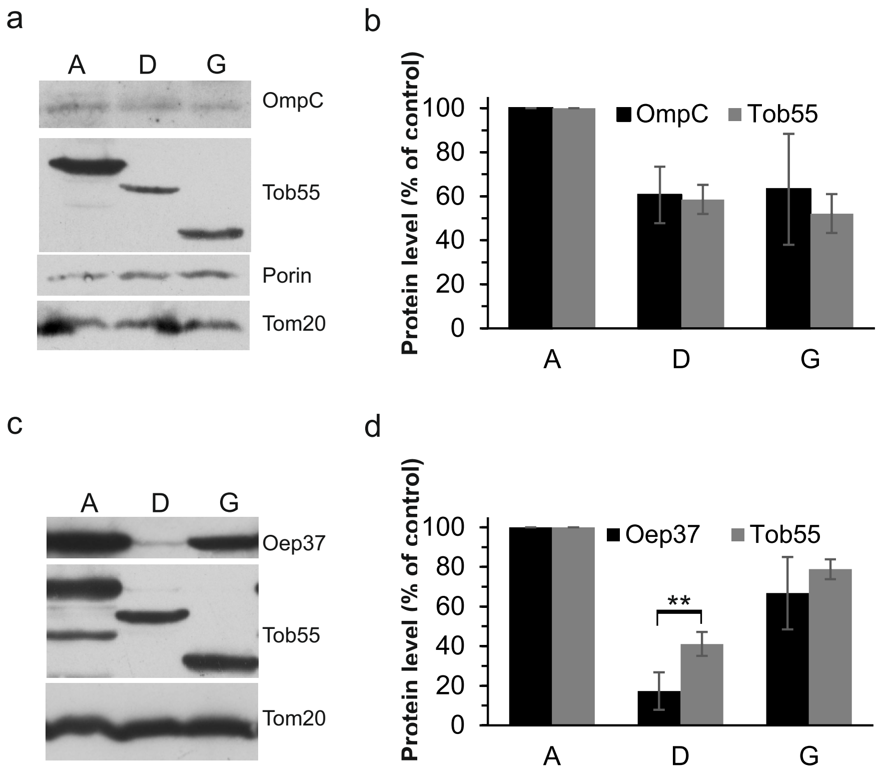

Figure S4: Steady-state levels of bacterial β-barrel protein OmpC and chloroplast β-barrel protein Oep37 are reduced in cells habouring Tob55-hybrids proteins. a. Proteins of crude mitochondria isolated from Tob55-depleted cells co-expressing the indicated hybrid protein and OmpC were analysed by SDS-PAGE followed by immunoblotting with antibodies against OmpC, Tob55, Porin (mitochondrial β-barrel protein) and Tom20 (single-span OM protein). **b**. Quantification of the bands of three independent experiments similar to A. Loading was normalised against Tom20 and intensitiy of Tob55-depleted cells transformed with Tob55 (hybrid A) was set to 100%. **c.** Crude mitochondria of Tob55-depleted cells co-expressing the indicated hybrid protein and Oep37 were analysed as described in (a). **d.** Quantification of three independent experiments similar to that depicted in (c) was performed as described in (b).

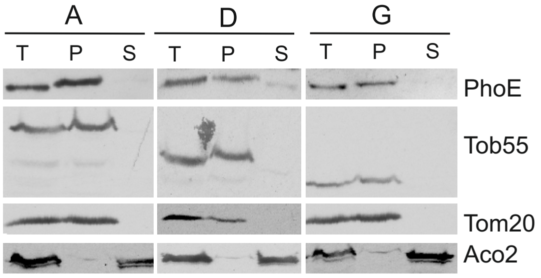

Figure S5: PhoE is membrane inserted in presence of Tob55/BamA variants D and G. Mitochondria from Tob55-depleted (GAL10Tob55) strains expressing the indicated Tob55/BamA variant and PhoE were isolated. Mitochondria (Total, T) were subjected to carbonate extraction separating membrane embedded proteins in the pellet (P) from soluble and membrane associated proteins in the supernatant (S). Proteins were analyzed by SDS/PAGE and immunodecoration with the indicated antibodies.

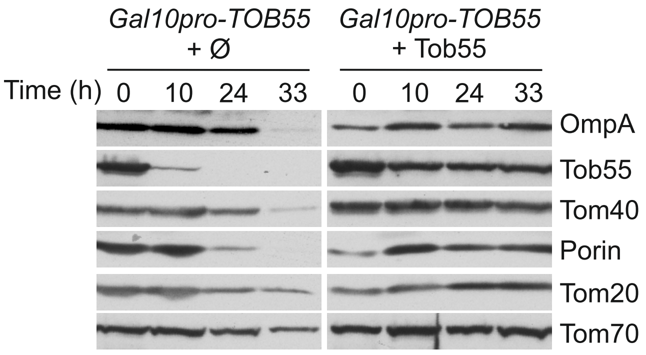

Figure S6: OmpA biogenesis is dependent on the TOB complex. Cells from a strain expressing Tob55 under the control of the *GAL10* promoter (Gal10pro-TOB55) were transformed with a plasmid encoding for OmpA and with a plasmid encoding for Tob55 or the respective empty plasmid. Cells were harvested at the indicated time points after shift to glucose-containing medium. Crude mitochondria were isolated and proteins were analyzed by SDS/PAGE and immunodecoration with antibodies against OmpA and the mitochondrial proteins Tob55, Tom40, and Porin (ß-barrel proteins), Tom20 and Tom70 (MOM proteins).

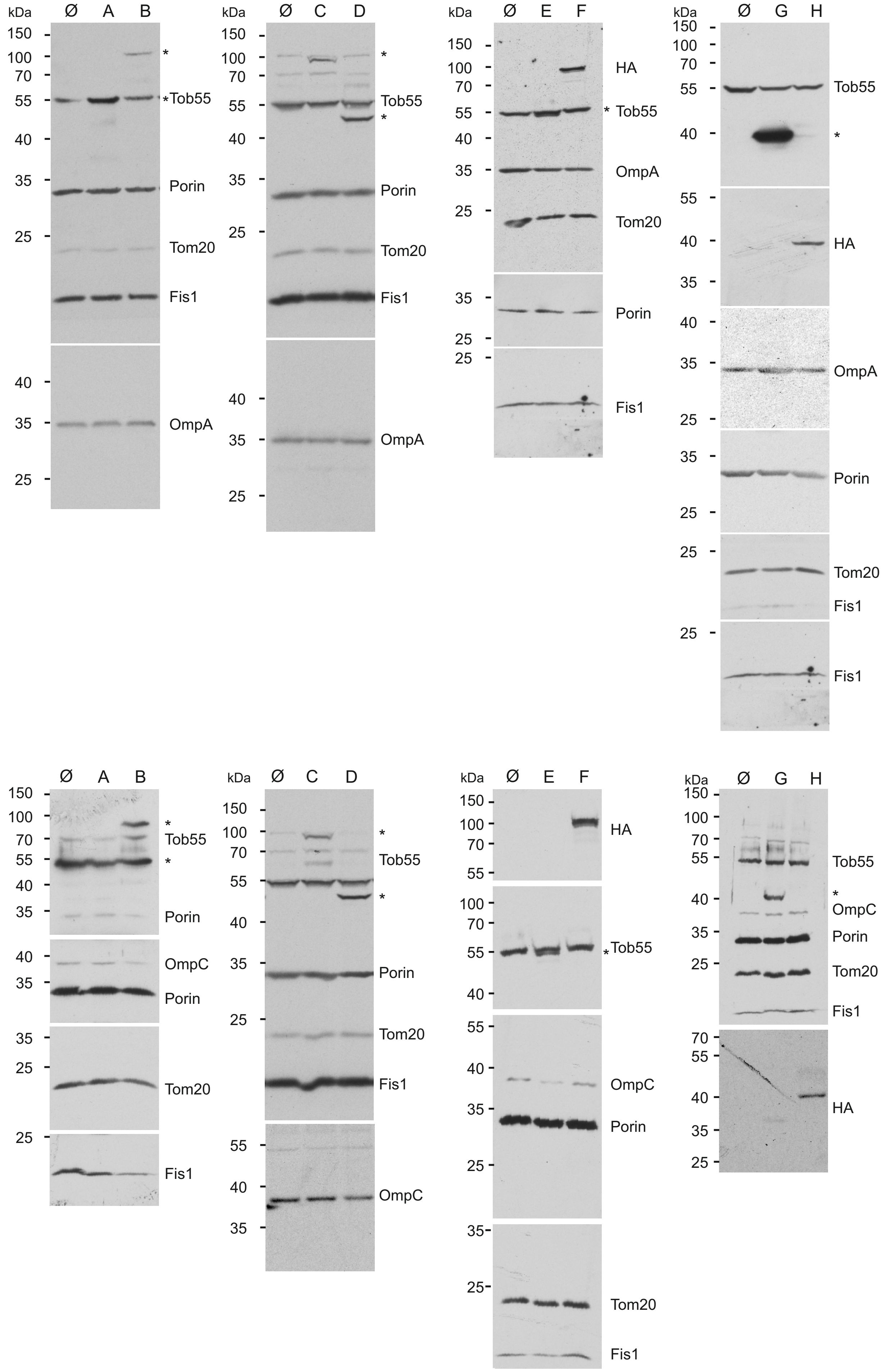

Supplementary Fig. 7. Uncropped immunodecorations of Fig. 1. Panels of OmpC and OmpA.

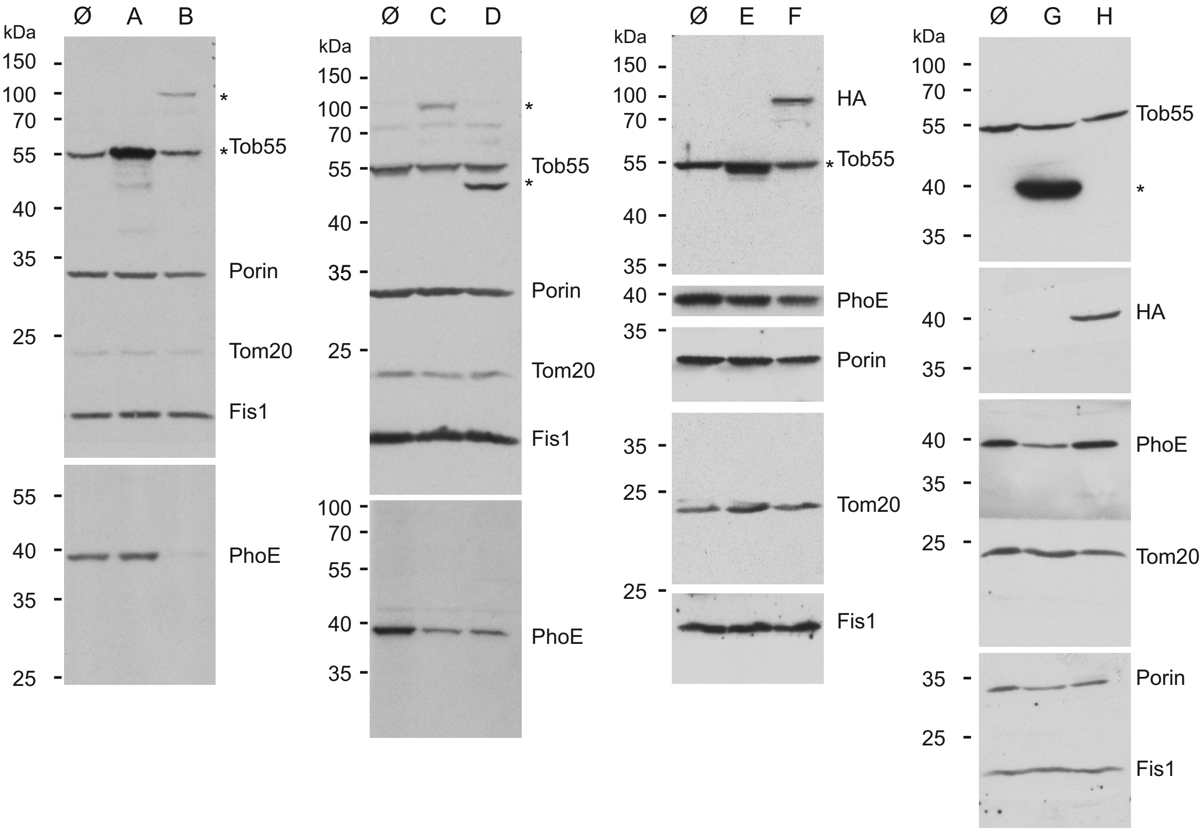

Supplementary Fig. 7. Uncropped immunodecorations of Fig. 1. Panels of PhoE.

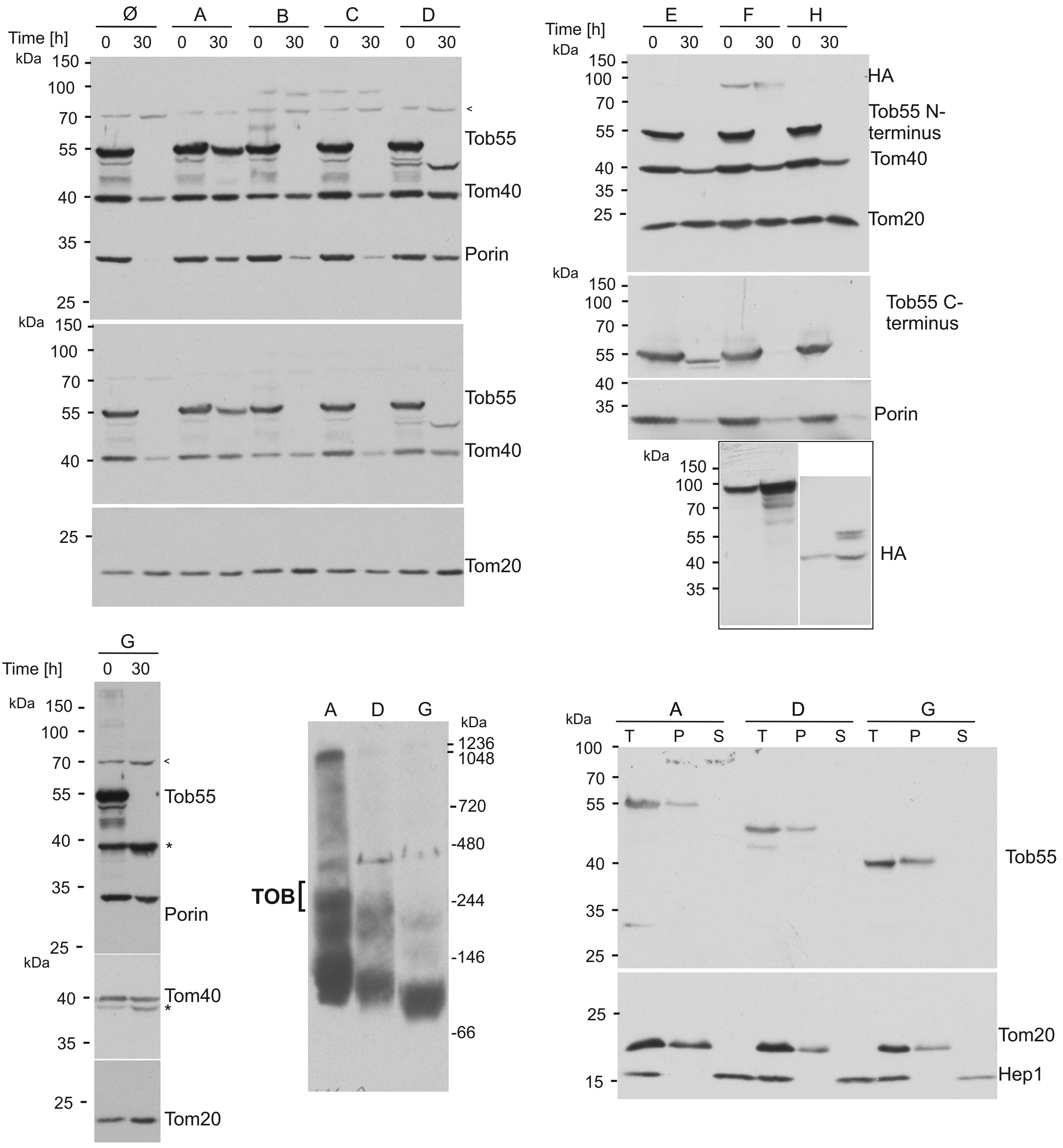

Supplementary Fig. 7. Uncropped immunodecorations of Fig. 2.

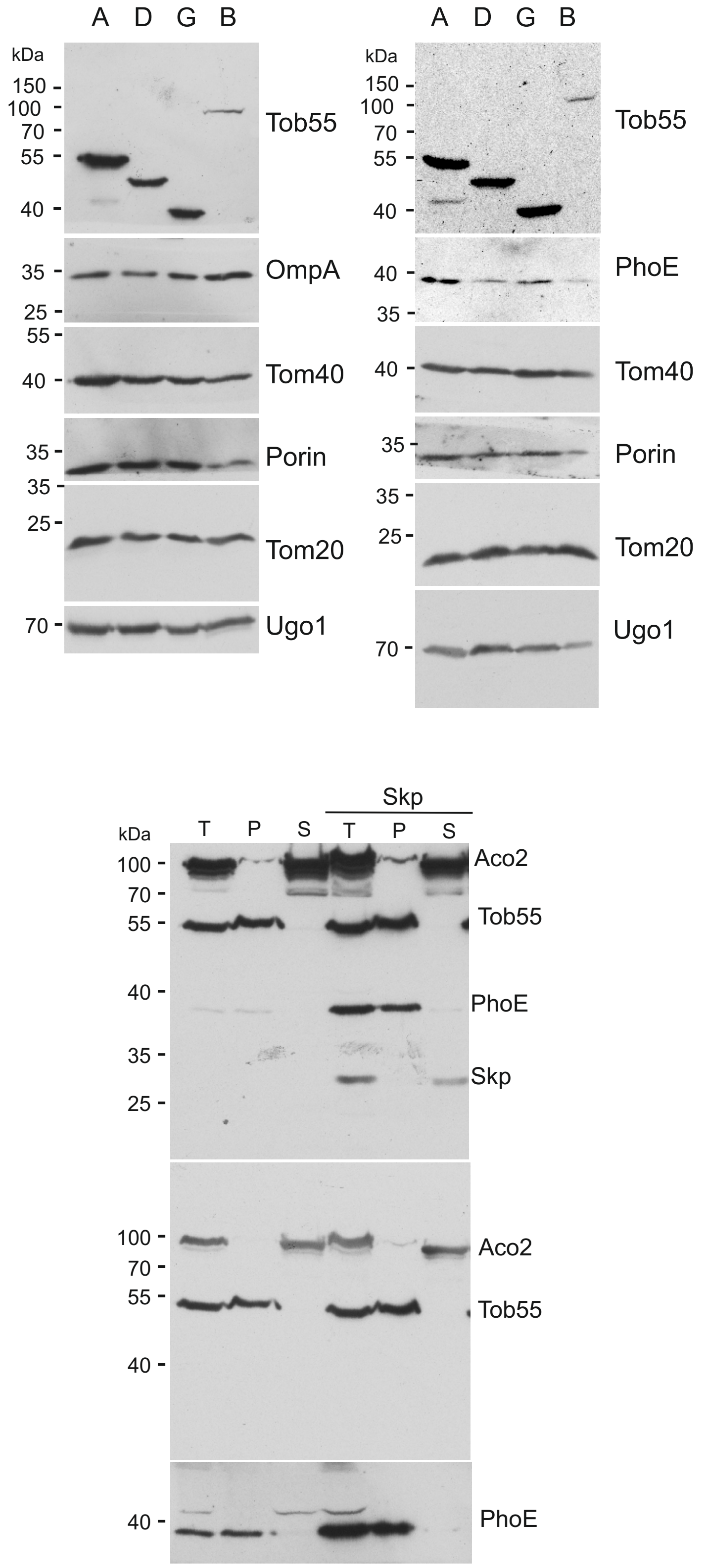

Supplementary Fig. 7. Uncropped immunodecorations of Figs. 4 and 5.

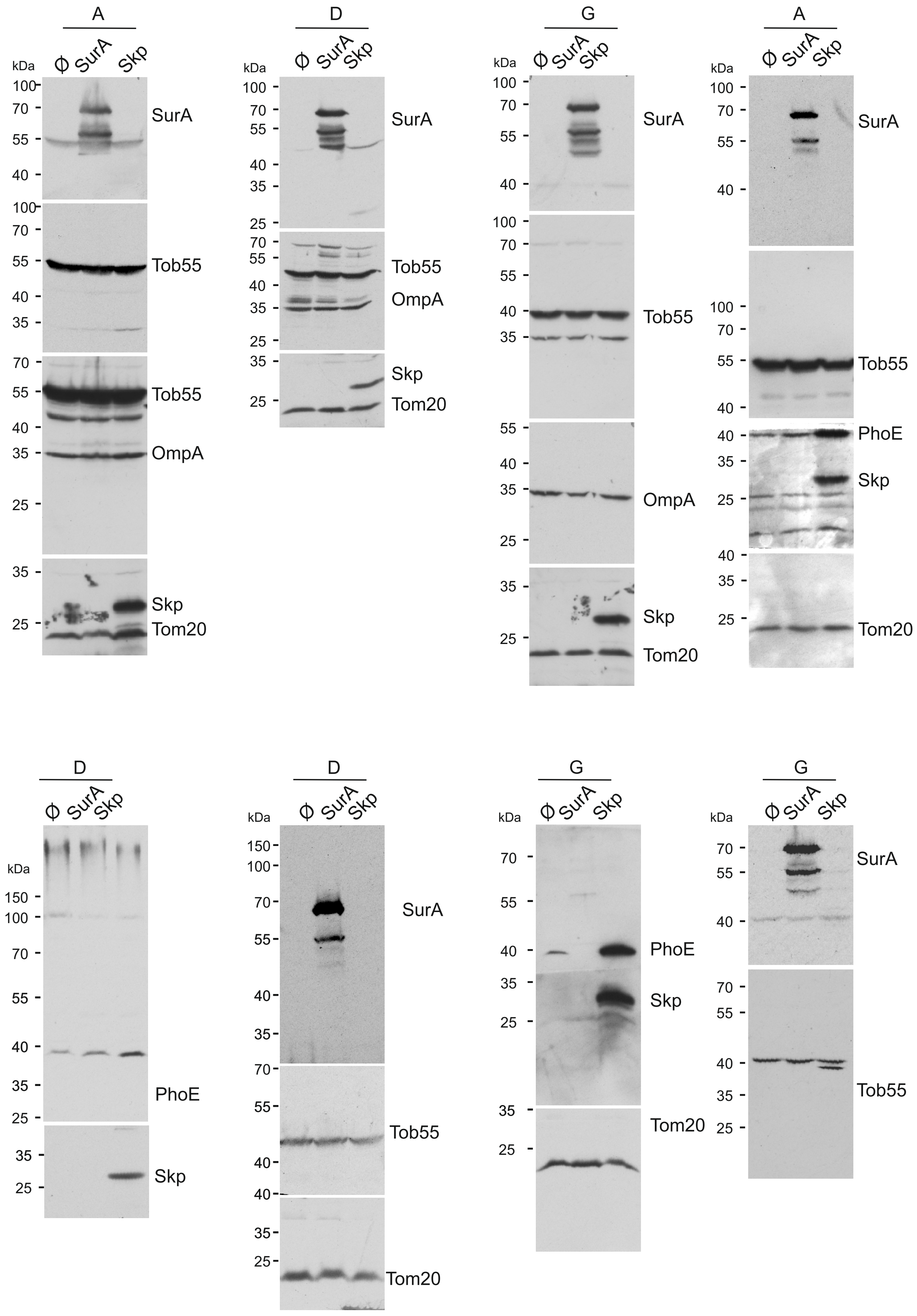

Supplementary Fig. 7. Uncropped immunodecorations of Fig. 5.

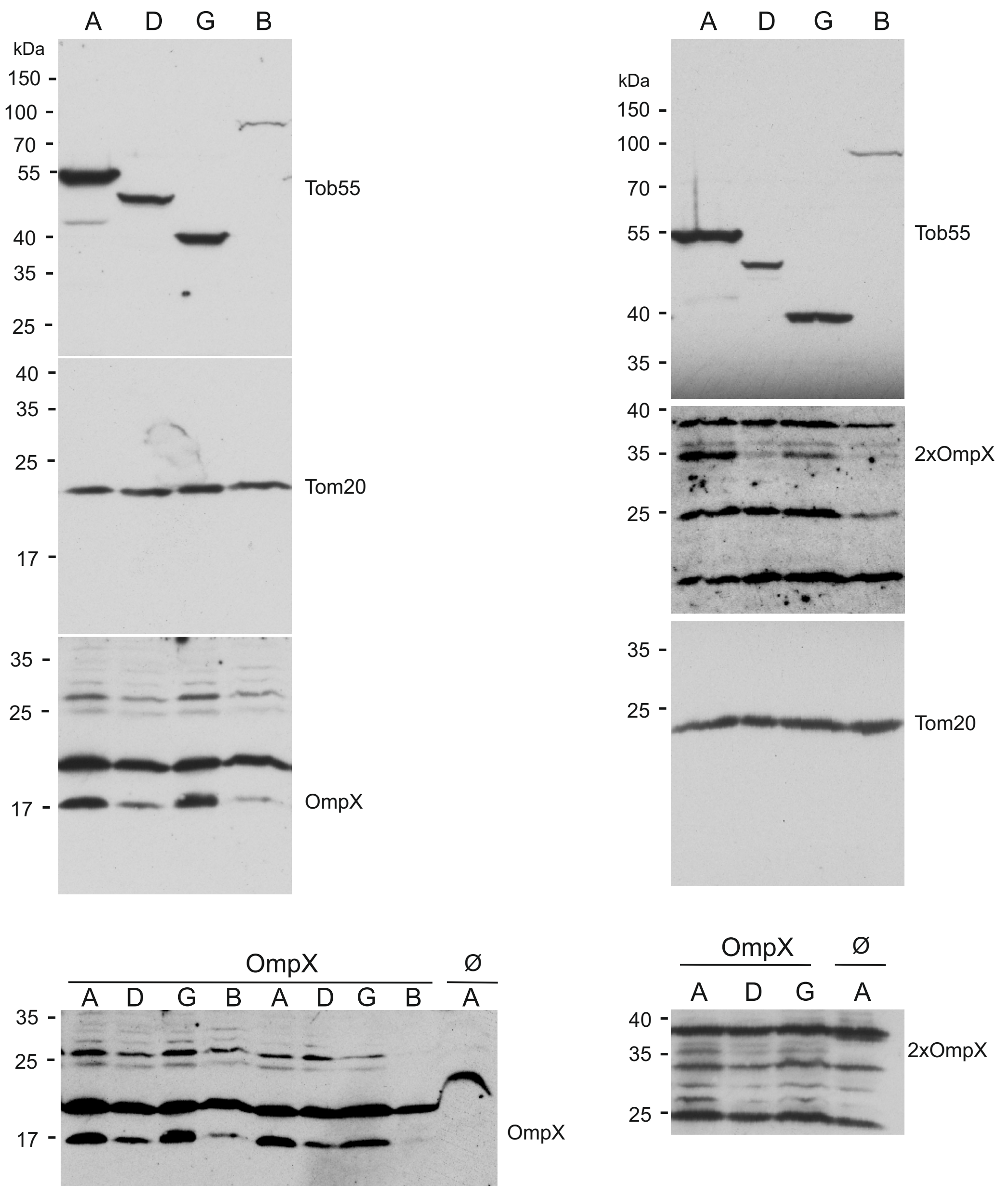

Supplementary Fig. 7. Uncropped immunodecorations of Fig. 6.

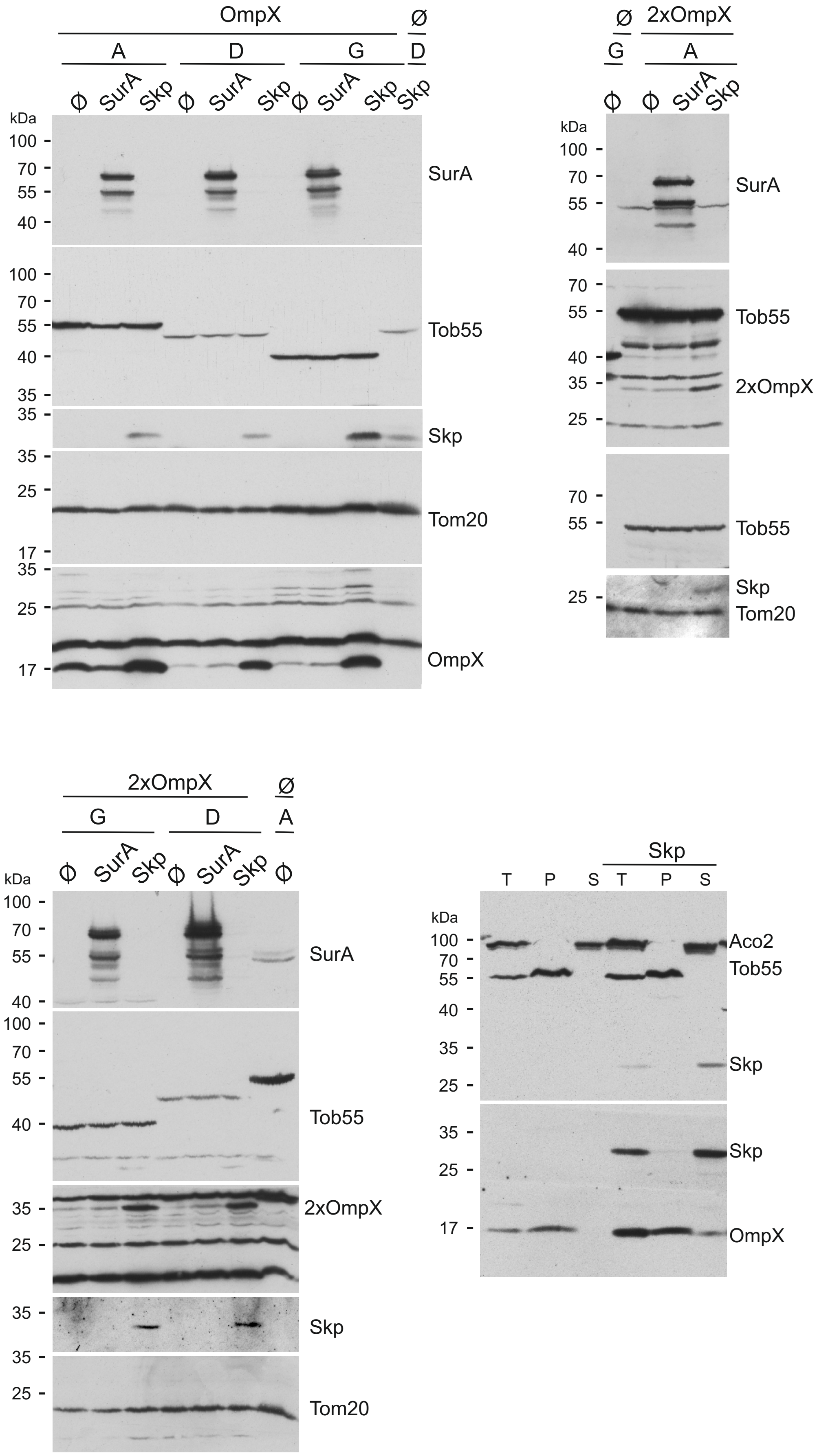

Supplementary Fig. 7. Uncropped immunodecorations of Fig. 6.

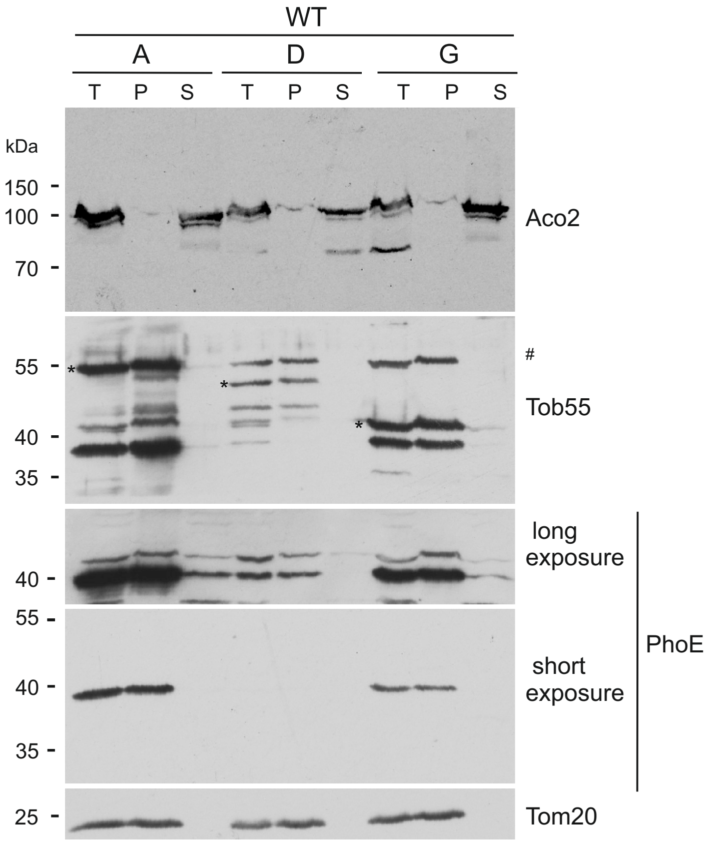

Supplementary Fig. 7. Uncropped immunodecorations of Supplementary Fig. S1.

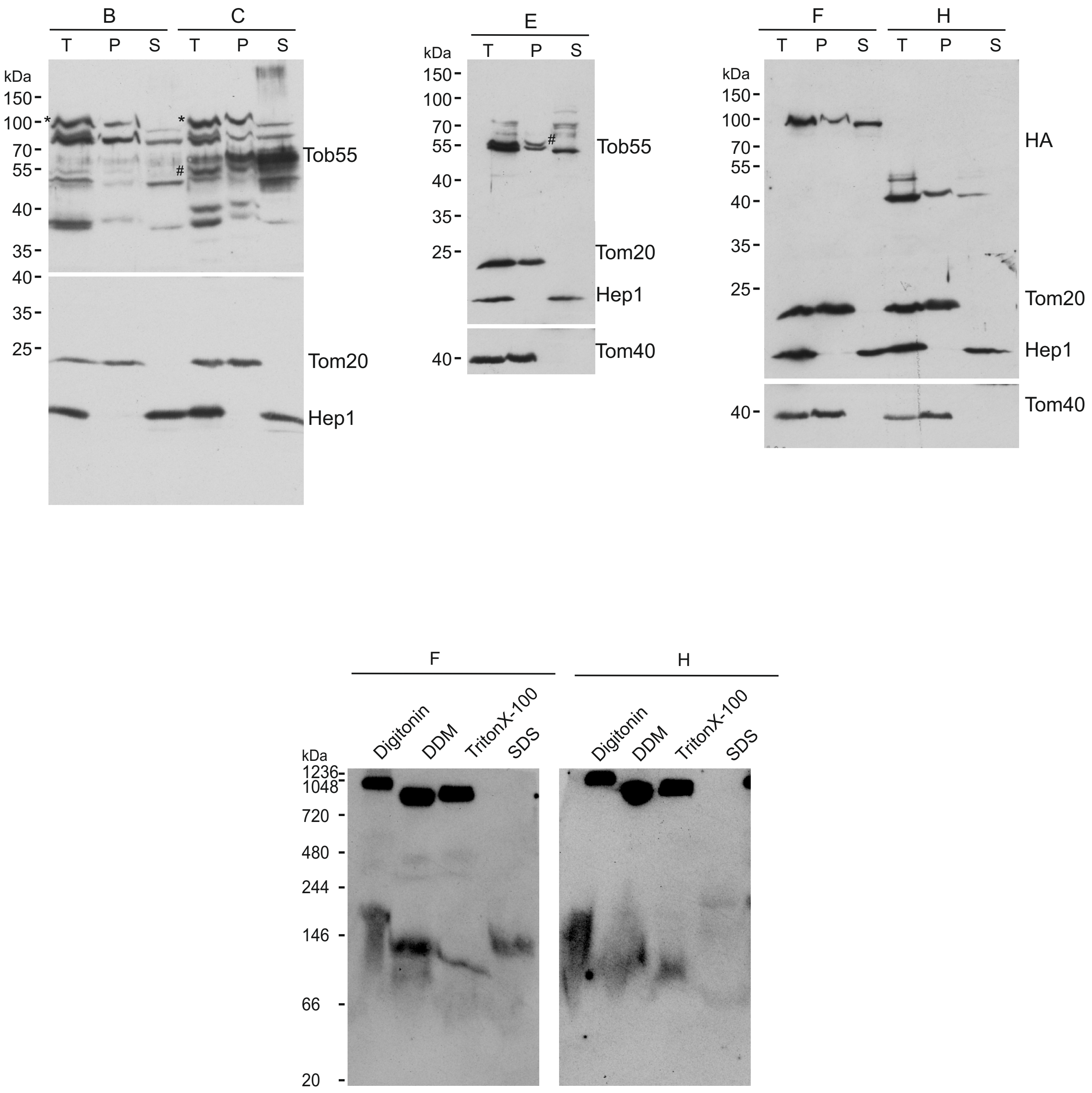

Supplementary Fig. 7. Uncropped immunodecorations of Supplementary Fig. S2.

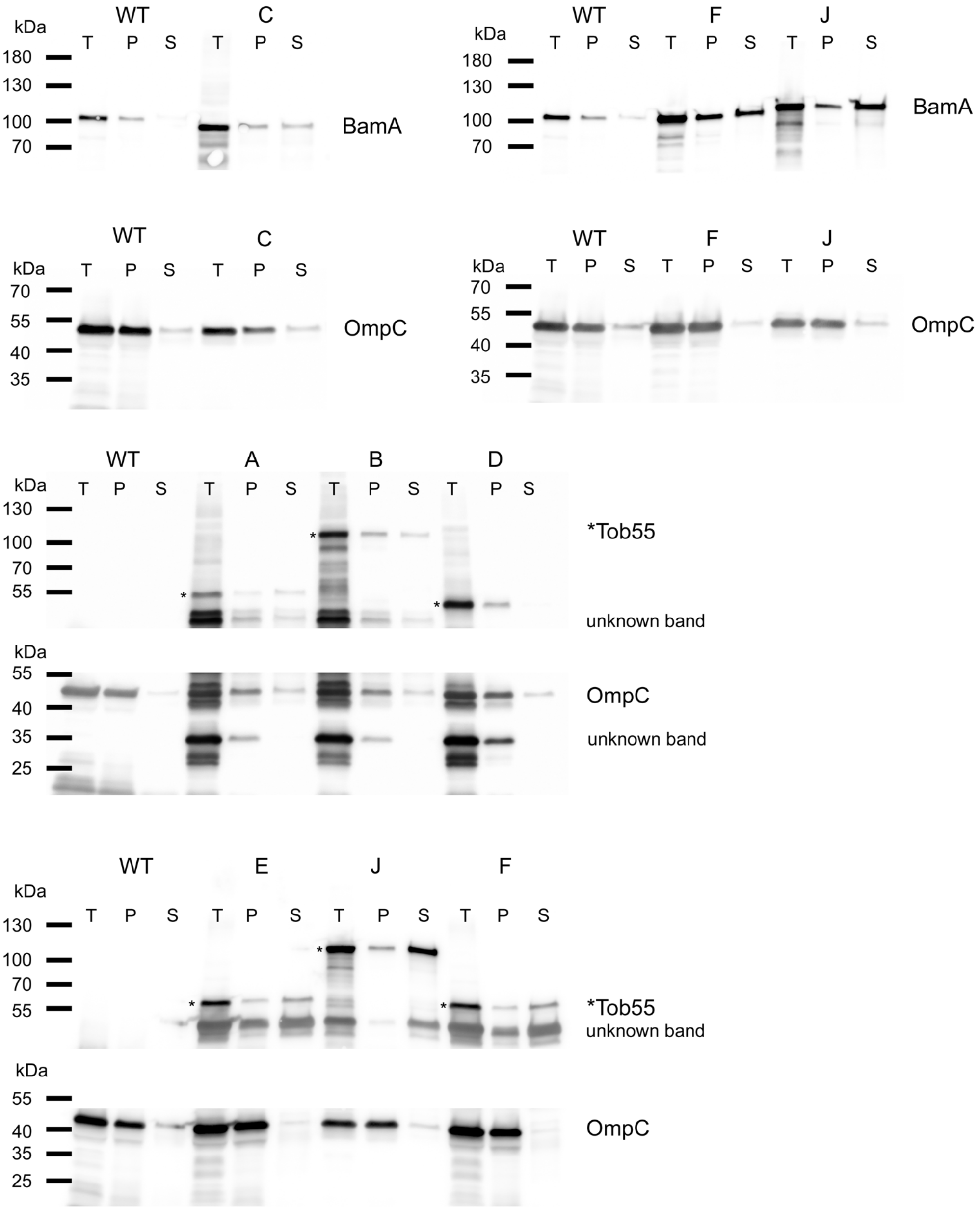

Supplementary Fig. 7. Uncropped immunodecorations of Supplementary Fig. S3.

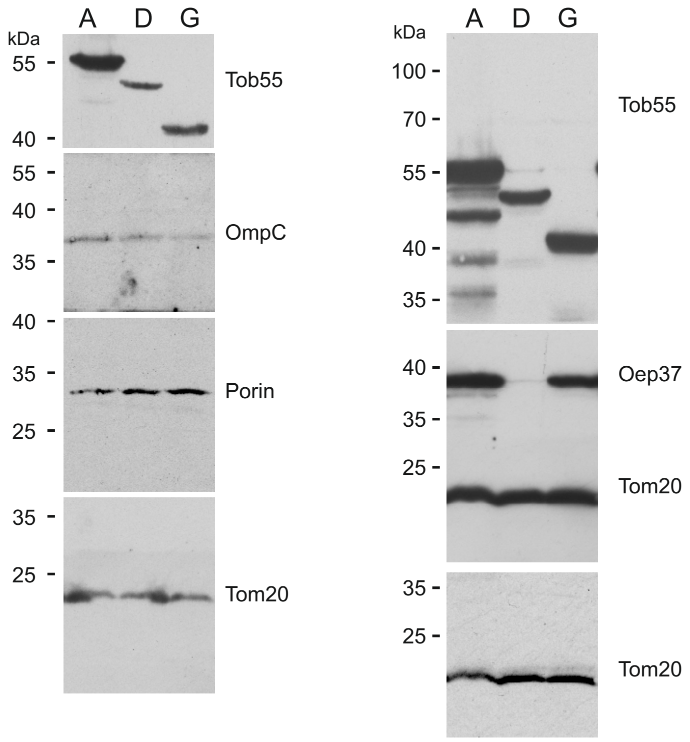

Supplementary Fig. 7. Uncropped immunodecorations of Supplementary Fig. S4.

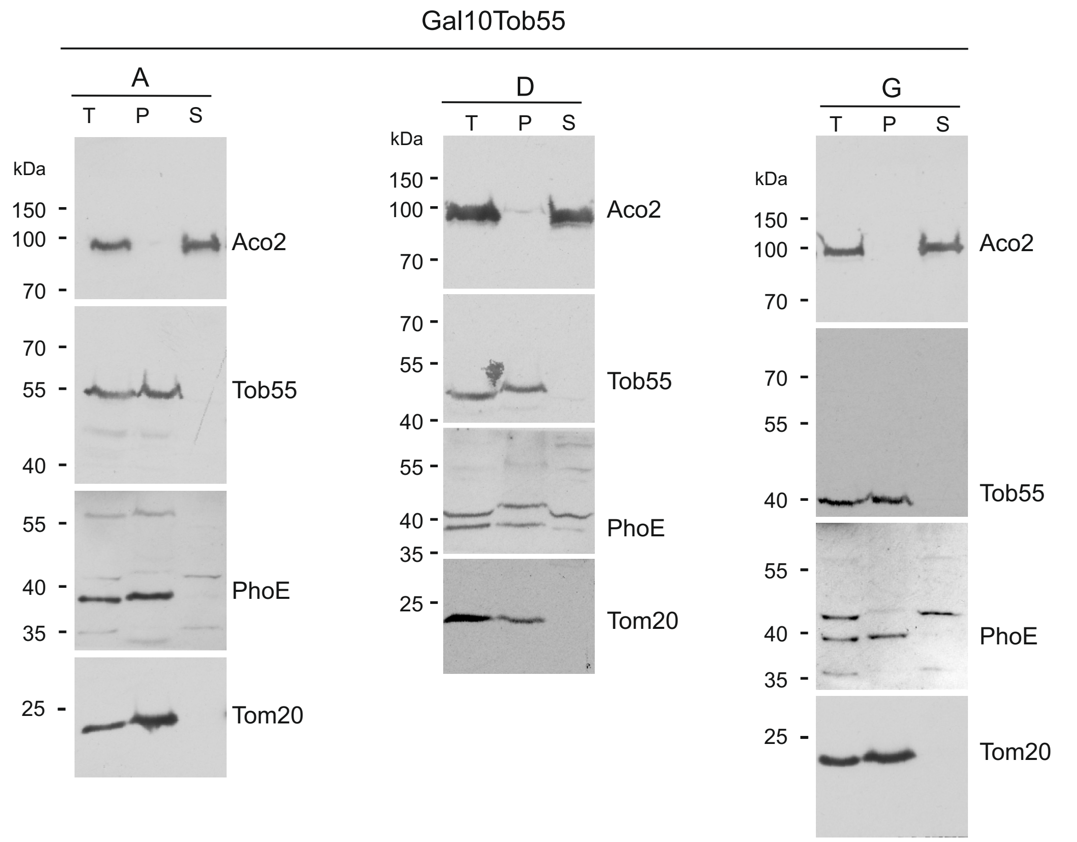

Supplementary Fig. 7. Uncropped immunodecorations of Supplementary Fig. S5.

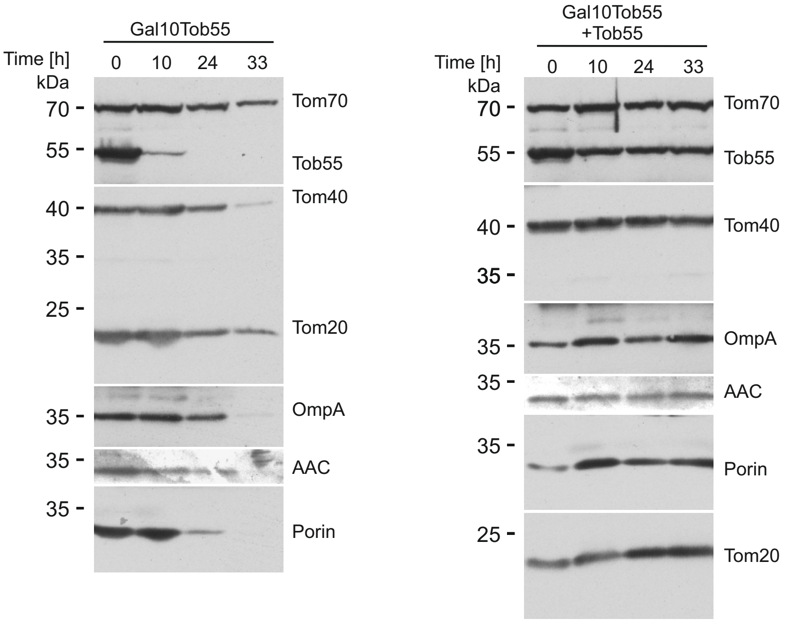

Supplementary Fig. 7. Uncropped immunodecorations of Supplementary Fig. S6.
